# The Role and Therapeutic Potential of the cGAS‐STING Signaling Pathway in Alzheimer's Disease

**DOI:** 10.1002/brb3.71130

**Published:** 2025-12-10

**Authors:** Xue Li, Wei Gao, Qiuyan Ye, Honglin Li

**Affiliations:** ^1^ Heilongjiang University of Chinese Medicine Harbin China; ^2^ Jiangsu College of Nursing Huaian China; ^3^ Department of Rehabilitation Medicine III The Second Affiliated Hospital of Heilongjiang University of Chinese Medicine Harbin China

**Keywords:** alzheimer's disease, cGAS, neuroinflammation, STING

## Abstract

**Purpose:**

Alzheimer's disease (AD) is a neurodegenerative disorder characterized by progressive cognitive decline, posing a significant challenge to global public health. As a core signaling pathway in the mammalian innate immune system, the cyclic GMP‐AMP synthase (cGAS)‐stimulator of interferon genes (STING) pathway plays a pivotal role in maintaining intracellular homeostasis. This review aims to systematically elucidate the role and therapeutic potential of the cGAS‐STING signaling pathway in AD, focusing on its involvement in key pathological processes and its relevance to AD risk factors.

**Method:**

Through literature search, we summarized the molecular mechanisms of the cGAS‐STING pathway and its dysregulation in AD, emphasizing the integrated evidence linking cGAS‐STING to neuroinflammation, autophagy impairment, and neuronal death, as well as its interactions with aging, obesity, cardiovascular disease, and diabetes.

**Findings:**

The cGAS‐STING pathway is critically involved in AD pathogenesis, contributing to neuroinflammation, defective autophagy, and neuronal loss. Its activation is associated with multiple AD risk factors, suggesting a broad influence on disease progression. Pharmacological inhibition of cGAS‐STING shows promise in attenuating these pathological features in preclinical models.

**Conclusion:**

The cGAS‐STING signaling pathway plays a central regulatory role in the central nervous system, and its dysregulation promotes neuroinflammation and is closely associated with AD. This pathway forms a vicious cycle by integrating multiple pathological signals, including mitochondrial dysfunction and endoplasmic reticulum stress. Small‐molecule inhibitors and natural products targeting this pathway have demonstrated significant efficacy in preclinical studies, providing a basis for developing disease‐modifying therapies for AD. Future efforts should focus on multi‐target combination strategies (e.g., STING inhibitors co‐administered with Aβ/tau drugs) and dynamically deciphering pathway alterations across AD stages to advance personalized treatment approaches.

## Graphical abstract

This work reveals the relationship between the cGAS‐STING signaling pathway and the pathological mechanisms of Alzheimer's disease.The role of the cGAS‐STING signaling pathway in AD pathology. AD leads to the production of neutrophil extracellular traps (NETs), endoplasmic reticulum (ER) stress, and the release of mitochondrial DNA (mtDNA) and nuclear DNA, which activate the cGAS‐STING signaling pathway, thereby exacerbating Aβ plaque deposition, neuroinflammation, pyroptosis, and autophagy.

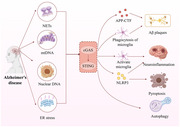

## 1 Introduction

Alzheimer's disease (AD), a prevalent form of senile dementia worldwide, is a neurodegenerative disorder characterized by progressive cognitive decline. This condition has consequently become a major focus of global public health efforts (Scheltens et al. 2021). Epidemiological studies indicate that as the global population ages, the prevalence of AD continues to rise, with projections suggesting the number of affected individuals will reach 152 million by 2050 (Nichols et al. 2021). This escalating prevalence profoundly diminishes the quality of life for patients and places a significant burden on healthcare infrastructures, social welfare programs, and familial caregivers. Therefore, it is critically important to decipher the molecular pathways responsible for AD pathogenesis and to pioneer novel treatment approaches.

The cyclic GMP‐AMP synthase (cGAS)‐stimulator of interferon genes (STING) signaling pathway serves as a fundamental hub for innate immunity. Within this system, the cytosolic DNA sensor cGAS identifies the presence of double‐stranded DNA (dsDNA), which can originate from damaged host cells (self‐DNA) or foreign pathogens (Liu et al. 2021). Triggered by cellular damage or viral infection, cGAS synthesizes the second messenger 2'3'‐cyclic GMP‐AMP (cGAMP) upon binding dsDNA, which in turn activates the STING protein. Once activated, STING propagates signals via the nuclear factor kappa B (NF‐κB) pathway and the TANK‐binding kinase 1 (TBK1)‐interferon regulatory factor 3 (IRF3) axis. These signaling events ultimately induce the secretion of type I interferons (IFNs) and proinflammatory cytokines, molecules that are essential for mounting an immune defense (Jia et al. 2024, Tesser et al. 2021). In addition to governing immunoinflammatory responses, this pathway is a key regulator of core cellular processes such as autophagy, senescence, and programmed cell death (Zhou et al. 2023, Gui et al. 2019). A growing body of research now highlights the significant implication of cGAS‐STING signaling in major neurodegenerative disorders, including AD (Xie et al. 2023), Parkinson's disease (Standaert and Childers 2022), Huntington's disease (Jauhari et al. 2020), and amyotrophic lateral sclerosis (Tan et al. 2022). This review systematically explores the molecular mechanisms of the cGAS‐STING pathway, details its contributions to AD onset and progression, and assesses recent advances in creating cGAS and STING pharmacological inhibitors. Our objective is to provide a comprehensive evaluation of the therapeutic potential in targeting this pathway for Alzheimer's disease.

## 2 Mechanism of the cGAS‐STING Pathway

As a cytoplasmic DNA sensor, cGAS belongs to the nucleotidyltransferase (NTase) family. Its architecture consists of three primary elements: an N‐terminal domain, a highly conserved C‐terminal NTase domain that acts as the catalytic core, and a Mab21 domain. While the N‐terminal domain is vital for protein stabilization and autoinhibition, the NTase domain is responsible for both DNA recognition and binding; it also contains an autoinhibitory surface that prevents spurious activation by self‐DNA (Chauhan and Kaundal 2023). In its basal state, cGAS remains enzymatically dormant and requires interaction with dsDNA to become active. The disruption of cellular homeostasis, which results in the accumulation of cytoplasmic DNA, activates cGAS through its binding to dsDNA (Wang et al. 2020). Subsequently, the activated cGAS catalyzes the synthesis of cyclic cGAMP, a second‐messenger molecule that binds to the adapter protein STING on the endoplasmic reticulum (ER) membrane. This binding induces the translocation of STING from the ER to the Golgi apparatus and ultimately triggers a downstream phosphorylation cascade involving TBK1 and IκB kinase (IKK) (Ablasser et al. 2013).

**FIGURE 1:**
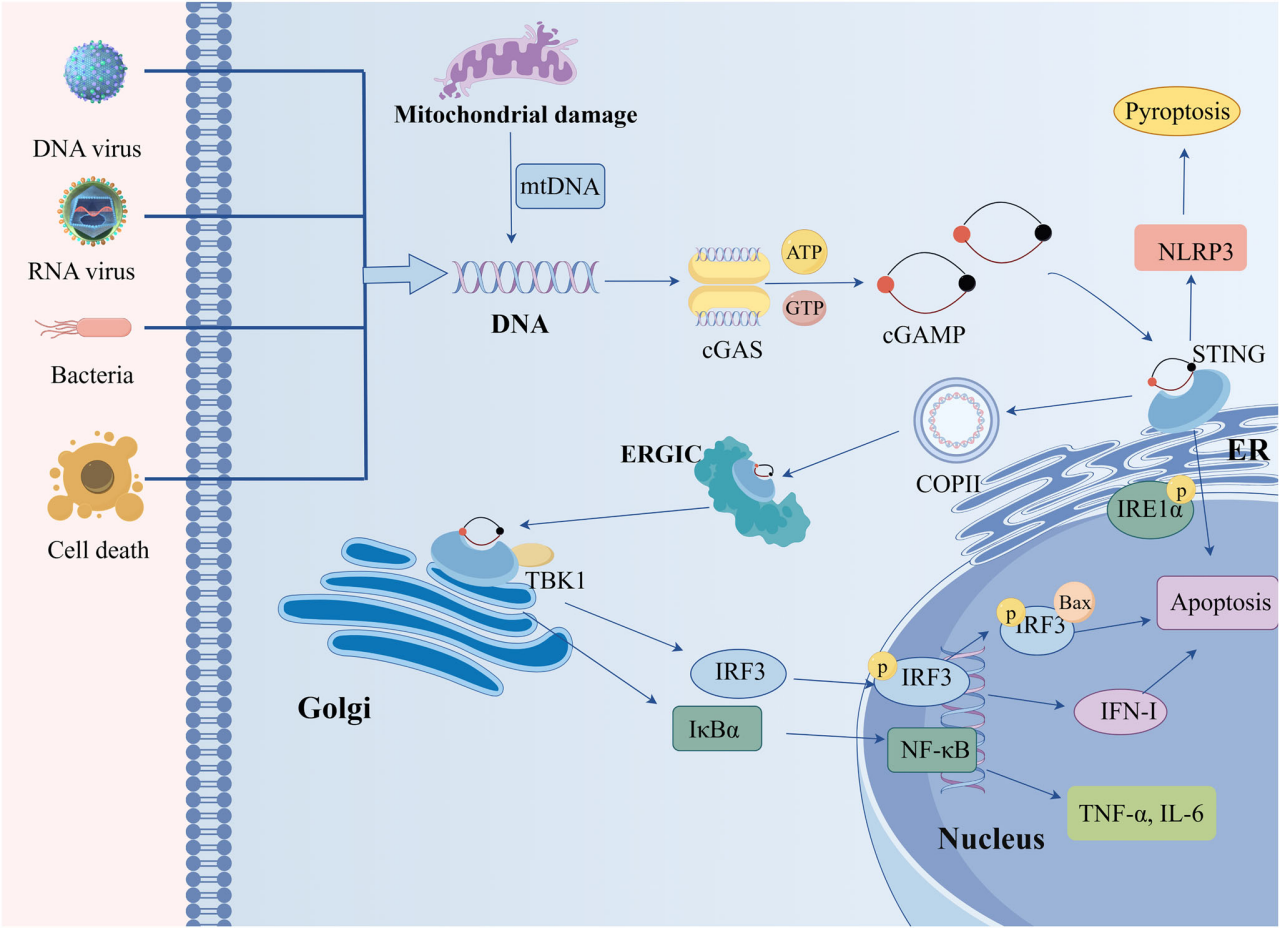
Overview of the cGAS‐STING pathway. When exogenous DNA invasion (such as DNA viruses) and cell damage lead to the accumulation of endogenous DNA, it binds to free cGAS in the cytoplasm. Activated cGAS synthesizes cGAMP through guanosine triphosphate (GTP) and adenosine triphosphate (ATP). cGAMP binds to STING on the endoplasmic reticulum (ER), promoting the transport of STING to the Golgi apparatus through COPII and the intermediate compartment of the ER‐Golgi apparatus (ERGIC), and facilitating the phosphorylation of TBK1. The phosphorylation of TBK1 exerts its function by promoting the migration of IRF3 and NF‐κB to the nucleus to trigger type I interferon transcription and activate inflammatory pathways. The cGAS‐STING signaling pathway can induce pyroptosis by activating NLRP3. STING signaling can promote apoptosis by facilitating the phosphorylation of IRE1α. In addition, phosphorylated IRF3 directly interacts with BAX to promote apoptosis (by Figdraw).

The subsequent activation of key effector proteins, IRF3 and NF‐κB, leads to the transcriptional upregulation of type IFNs and various proinflammatory cytokines, thereby launching an immune defense (Yang et al. 2024, Long et al. 2022). Apart from this well‐characterized signaling cascade, the cGAS‐STING pathway also contributes to a wide array of other biological functions. These include processes regulated by protein kinase R‐like ER kinase (PERK), signal transducer and activator of transcription 6 (STAT6), as well as oxidative stress, apoptosis, autophagy, and inflammasome activation (Lv et al. 2023). In the context of autophagy, cGAS binds to the autophagy‐initiating protein Beclin‐1, which suppresses cGAMP synthesis and facilitates the degradation of cytoplasmic DNA, thereby preventing excessive immune activation (Liang et al. 2014). Furthermore, STING itself can directly induce autophagy to modulate innate immune responses (Liu et al. 2019). The degradation of STING, in turn, is mediated by the selective autophagy receptor p62, which traffics ubiquitinated STING to autophagosomes for degradation, a process that suppresses interferon production (Prabakaran et al. 2018). Regarding pyroptosis, the cGAS‐STING pathway drives this process by activating NLRP3 inflammasomes, leading to gasdermin‐mediated pyroptosis. For example, in nucleus pulposus cells and neutrophils, mitochondrial DNA leakage activates the cGAS‐STING pathway, which promotes NLRP3 inflammasome activation and induces pyroptosis (Zhang et al. 2022). Mechanistically, STING promotes NLRP3 activation either by binding to it within the endoplasmic reticulum to facilitate its oligomerization, or by enhancing NLRP3 expression via deubiquitination and epigenetic regulation (Ming et al. 2020, Wang et al. 2020) (Figure 1).

## 3 Role of cGAS‐STING Pathway in the Risk Factors of AD

### 3.1 Aging

Aging is a primary risk factor for AD, correlating strongly with a marked increase in its prevalence; the majority of AD cases are late‐onset, typically manifesting after the age of 65 (Liu 2022). In the United States, estimates indicate a prevalence of AD as high as 11% in adults aged 65 and older, rising to approximately 32% in those aged 85 and older (Dintica and Yaffe 2022). This age‐related susceptibility is closely linked to progressive structural changes in the brain, including cerebral volume reduction, decreased synaptic density, ventricular dilatation, and the accumulation of pathological hallmarks such as amyloid plaques and neurofibrillary tangles (Hou et al. 2019). Cellular senescence is a key driver of physiological aging and is implicated in numerous age‐related diseases (Kumari and Jat 2021). During cellular senescence, the cGAS‐STING pathway drives disease progression through a defined sequence: damage signal recognition, inflammation triggering, and exacerbation of AD pathology. First, in aging mouse models, cGAS recognizes cytoplasmic chromatin fragments (CCFs) released by senescent cells. STING activation subsequently induces the senescence‐associated secretory phenotype (SASP), leading to the release of pro‐inflammatory factors such as IL‐1β and TNFα (Dou et al. 2017, Passarella et al. 2024). These SASP factors activate astrocytes, impairing their phagocytic capacity for Aβ and upregulating the expression of Aβ‐generating enzymes like BACE1, which collectively accelerates Aβ deposition (Kong et al. 2020). Second, mitochondrial dysfunction in microglia—the brain's resident immune cells—leads to mitochondrial DNA (mtDNA) release, which activates the cGAS‐STING pathway and drives their transformation into a disease‐associated microglia (DAM) phenotype (Gulen et al. 2023, Tan et al. 2022). This DAM phenotype is characterized by a significant reduction in the clearance of both Aβ and pathological tau, coupled with the release of pro‐inflammatory factors that promote excessive tau phosphorylation in neurons. This leads to the formation of neurofibrillary tangles (NFTs), neuronal death, and cognitive impairment (Lin et al. 2021). Furthermore, in neurodegenerative diseases, immune responses to DNA double‐strand breaks (DSBs), mediated by NF‐κB, are a major driver of brain aging. When neuronal genomic stability is compromised, cGAS expression is upregulated, and it acts as an upstream regulator to activate the NF‐κB pathway, a mechanism validated in AD animal models (Welch et al. 2022).

### 3.2 Obesity

Obesity, a complex metabolic disorder characterized by excessive adipose tissue accumulation, exerts pathological effects that extend beyond metabolic dysregulation. Dysfunctional adipokine signaling and impaired adipocyte physiology are central to its pathogenesis and significantly elevate the risk of neurodegenerative diseases, including AD (Uddin et al. 2020). Notably, the association between obesity and AD is age‐dependent. Midlife obesity represents a significant risk factor that can trigger AD‐related neuropathological changes years before clinical dementia symptoms manifest (Lee 2011, Moser and Pike 2016). Conversely, overweight status in later life is associated with a reduced risk of AD, mild cognitive impairment (MCI), and vascular dementia (Doruk et al. 2010).

The expansion of adipose tissue in obesity worsens AD pathology through multiple mechanisms: On the one hand, it promotes a state of chronic low‐grade inflammation that, together with oxidative stress, contributes to mitochondrial membrane disruption and genomic instability, ultimately resulting in mitochondrial impairment. The release of mtDNA from injured mitochondria can initiate cGAS‐STING signaling, creating a self‐sustaining cycle that reinforces sterile inflammation (Wu et al. 2019, Bai et al. 2017). Pro‐inflammatory cytokines such as IFN‐β, produced by activated peripheral immune cells, can traverse the blood‐brain barrier to activate the cGAS‐STING pathway in brain microglia, thereby promoting their M1 polarization (Kong et al. 2022, Preeti et al. 2024). Subsequently, IL‐6 released by these M1 microglia disrupts neuronal synaptic structures and suppresses the expression of Aβ clearance proteins such as LRP1, thereby exacerbating Aβ accumulation (Ham et al. 2019, Yang et al. 2016). Conversely, in the context of obesity, a deficiency in the adipose tissue protein DsbA‐L and excessive fatty acid accumulation activate the cGAS‐STING pathway by increasing mitochondrial ROS and promoting ceramide synthesis, respectively (Cruz et al. 2018, Huang et al. 2025). Ceramides further exacerbate mtDNA release by altering mitochondrial permeability and concurrently inhibit central insulin signaling pathways. This leads to cerebral insulin resistance, which ultimately promotes tau phosphorylation and neuronal death (Xu et al. 2020).

### 3.3 Cardiovascular Diseases

Cardiovascular disease (CVD), a leading global cause of disability and mortality, encompasses a spectrum of conditions, including heart failure, myocardial infarction, and coronary artery disease. Its high incidence and mortality rates impose a substantial burden on both public health and socioeconomic systems (Zhang et al. 2023). Epidemiological studies have established a strong pathological link between CVD and AD, indicating that CVD patients face a significantly elevated risk of developing AD compared to the general population (Tublin et al. 2019, Wu et al. 2016). CVD contributes to the progression of AD pathology by activating the cGAS‐STING pathway in the brain through vasogenic injury. Notably, in atherosclerosis, damaged endothelial cells and macrophages release DNA fragments and other damage‐associated molecular patterns (DAMPs), which activate the cGAS‐STING pathway and upregulate IRF3 expression (Rech and Rainer 2021, Liu et al. 2017). A similar activation of the cGAS‐STING‐TBK1‐IRF3 signaling axis occurs in cardiomyocytes during myocardial ischemia‐reperfusion injury (Zhai et al. 2024). Activated IRF3 drives the release of proinflammatory cytokines, including IFN‐β, TNF‐α, and IL‐6. These cytokines induce a proinflammatory phenotype in glial cells, exacerbating neuroinflammation and synaptic damage. Concurrently, IRF3 directly regulates the expression of AD‐associated genes such as Apoe and downstream targets like ZBP1, thereby promoting amyloid plaque‐related pathology (Joshi et al. 2024).

### 3.4 Diabetes

**FIGURE 2:**
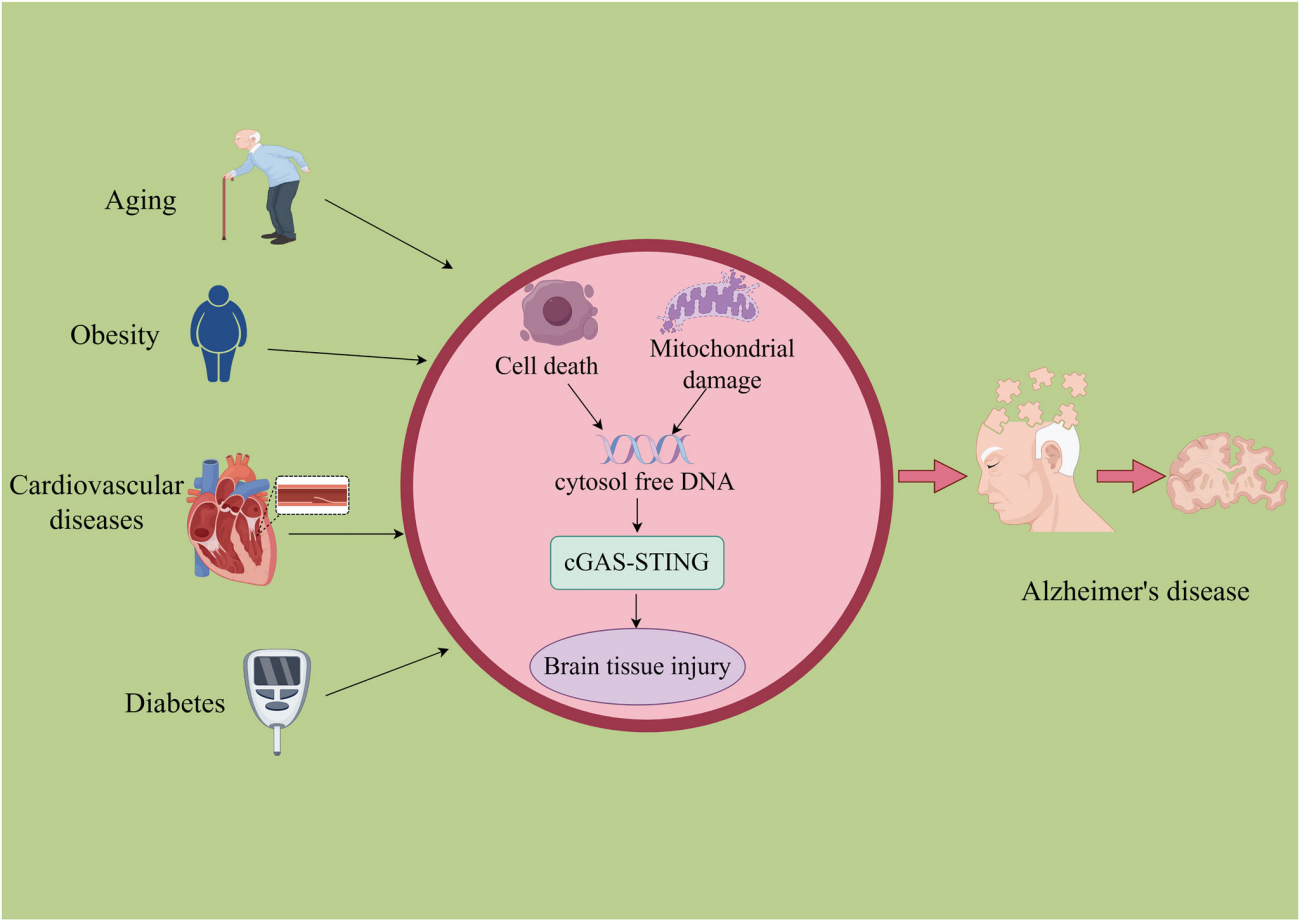
Risk factors of AD influence the cGAS‐STING pathway (by Figdraw).

Diabetes, an endocrine and metabolic disorder characterized by chronic hyperglycemia, is among the fastest‐growing chronic diseases worldwide. The global number of adult diabetic patients is projected to reach 693 million by 2045 (Cole and Florez 2020). Epidemiological studies indicate that elderly diabetic patients are more susceptible to widespread vascular lesions and face a significantly elevated risk of developing AD compared to non‐diabetic individuals (Biessels et al. 2006, Ahtiluoto et al. 2010). Diabetes promotes AD pathology by activating the cGAS‐STING pathway via mitochondrial damage. Initially, diabetes‐associated metabolic stress induces mitochondrial dysfunction in the brain, resulting in the leakage of mtDNA into the cytoplasm. This cytoplasmic mtDNA acts as a DAMP, activating the cGAS‐STING innate immune pathway and driving a robust type I interferon response, notably IFN‐β production. This cytokine response activates microglia and promotes their polarization toward a pro‐inflammatory (M1) phenotype, which triggers chronic neuroinflammation. These events ultimately lead to synaptic protein loss and the development of tau pathology, thereby impairing cognitive function (Preeti et al. 2024). Separately, free fatty acid‐induced mitochondrial oxidative damage also causes mtDNA leakage and cGAS‐STING pathway activation. In peripheral systems, this activation promotes proinflammatory responses and cardiomyocyte pyroptosis through NLRP3 inflammasome‐dependent mechanisms (Yan et al. 2022). NLRP3 inflammasome‐mediated pyroptosis can indirectly exacerbate neuroinflammation and directly activate tau‐phosphorylating kinases such as GSK‐3β. This increases the formation of NFTs, leading to neuronal death and cognitive decline (Xia et al. 2022, Zhu et al. 2021) (Figure 2) (Table 1).

**TABLE 1:** Associations between AD risk factors and the cGAS‐STING pathway.

Risk factor	cGAS‐STING activation triggers	Core mechanisms linking to AD pathology	Main effector cells	References
Aging	1. Senescent cells release cytoplasmic chromatin fragments (CCFs); 2. Microglial mitochondrial dysfunction releases mtDNA; 3. Neuronal DNA double‐strand breaks (DSBs)	1. SASP releases pro‐inflammatory factors that activate astrocytes, promote BACE1 expression, and accelerate Aβ deposition; 2. DAM microglia exhibit reduced clearance of Aβ/tau and induce excessive tau phosphorylation, leading to NFT formation; 3. NF‐κB pathway activation exacerbates neuroinflammation.	Astrocytes, Microglia, Neurons	(Dou et al. 2017, Passarella et al. 2024, Kong et al. 2020, Gulen et al. 2023, Welch et al. 2022)
Obesity	1. Adipose tissue expansion leads to mitochondrial damage and mtDNA release; 2. DsbA‐L deficiency increases mitochondrial ROS; 3. Excessive fatty acid accumulation promotes ceramide synthesis.	1. Peripheral factors like IFN‐β enter the brain, induce microglial M1 polarization, and suppress Aβ clearance; 2. Ceramides exacerbate mtDNA release, induce cerebral insulin resistance, and promote tau phosphorylation.	Peripheral Immune Cells, Microglia	(Wu et al. 2019, Preeti et al. 2024, Ham et al. 2019, Cruz et al. 2018, Huang et al. 2025)
Cardiovascular Disease (CVD)	1. Atherosclerotic lesions release nuclear DNA fragments and other DAMPs; 2. DNA release associated with myocardial ischemia‐reperfusion injury.	1. Activated IRF3 drives the release of IFN‐β, TNF‐α, etc., inducing a pro‐inflammatory phenotype in microglia, exacerbating neuroinflammation and synaptic damage; 2. Regulates the expression of Apoe and ZBP1, promoting Aβ deposition and neuroinflammation.	Vascular Endothelial Cells, Macrophages, Microglia	(Rech and Rainer 2021, Liu et al. 2017, Zhai et al. 2024, Joshi et al. 2024)
Diabetes	1. Metabolic stress causes brain mitochondrial dysfunction and mtDNA leakage; 2. Free fatty acid‐induced mitochondrial oxidative damage causes mtDNA escape.	1. IFN‐β activation promotes microglial pro‐inflammatory polarization, triggering neuroinflammation, synaptic loss, and tau pathology; 2. NLRP3 inflammasome‐mediated pyroptosis exacerbates brain inflammation, and GSK‐3β activation promotes NFT formation.	Microglia, Cardiomyocytes, Neurons	(Preeti et al. 2024, Cole and Florez 2020, Yan et al. 2022, Xia et al. 2022)

## 4 The Source of Activating the cGAS‐STING Signaling Pathway in AD

### 4.1 Neutrophil Extracellular Traps

Neutrophil extracellular traps (NETs) are web‐like complexes released by stimulated neutrophils, primarily consisting of DNA, histones, and antimicrobial proteins derived from granules (Zuo et al. 2020). The migration of neutrophils into the central nervous system (CNS) is a central pathological hallmark of numerous neuroinflammatory disorders (Rossi et al. 2011). Importantly, such neutrophil infiltration is strongly implicated in the progression of AD and the accompanying cognitive decline (Zenaro et al. 2015). Clinical investigations have revealed that circulating NET markers are substantially higher in the plasma and serum of AD patients relative to cognitively normal older adults (Kretzschmar et al. 2021). In animal models of AD, reducing neutrophil levels—either by depletion or by blocking neutrophil migration through LFA‐1 inhibition—significantly improves memory performance and lessens neuropathology (Pietronigro et al. 2017). Together, these observations underscore the active role of neutrophils and NETs in mediating tissue injury in AD. The dsDNA within NETs can be detected by the cytosolic DNA sensor cGAS, resulting in STING activation and the triggering of downstream signaling. NETs have been demonstrated to stimulate the cGAS‐STING pathway, enhancing the secretion of type I IFNs and proinflammatory cytokines, which amplifies immune activation. In contrast, impairment of key enzymes such as deoxyribonuclease I (DNase I) or peptidylarginine deiminase 4 (PAD4), which are essential for NET generation and preservation, suppresses cGAS‐STING signaling (Wang et al. 2021). Additionally, studies in a mouse model of traumatic brain injury (TBI) revealed that NETs induce ER stress through STING activation, aggravating neuroinflammation and programmed neuronal death (Shi et al. 2023). In conclusion, NETs serve as a major endogenous source of ligands that trigger cGAS‐STING signaling under neuroinflammatory conditions.

### 4.2 Mitochondrial DNA

mtDNA is a small, double‐stranded circular molecule enclosed within the double‐membrane system formed by the outer and inner mitochondrial membranes (OMM and IMM). The release of mtDNA into the cytosol has emerged as a central event in innate immune activation, triggering cGAS‐STING signal transduction (Kim et al. 2023). Under physiological conditions, mtDNA is strictly sequestered within the mitochondrial matrix by the OMM and IMM, preventing its recognition by cytosolic pattern recognition receptors (Li et al. 2024). However, upon cellular stress or mitochondrial dysfunction, mtDNA can translocate into the cytosol or extracellular space through mechanisms involving membrane damage or active transport. The mitochondrial protein transcription factor A (TFAM) facilitates cGAS dimerization by inducing DNA bending, thereby enhancing cGAS sensitivity to long DNA strands such as mtDNA. This TFAM‐mtDNA complex potently activates the cGAS‐STING pathway, inducing the production of proinflammatory cytokines and IFNs (Li et al. 2022, Lei et al. 2023). Mitochondrial dysfunction is a core driver of AD pathogenesis. For instance, the accumulation of APP‐CTF triggers mitochondrial structural alterations, overproduction of mitochondrial reactive oxygen species (mtROS), and impaired mitophagy in both AD mouse models and human brains (Vaillant‐Beuchot et al. 2021). During aging, exacerbated mtDNA oxidative damage acts synergistically with the secretion of SASP factors to promote inflammation (Li et al. 2024). Consequently, abnormal mtDNA dynamics during AD progression warrant particular attention. First, Hou et al. (Hou et al. 2021) observed a 3 to 6‐fold increase in cytosolic mtDNA enrichment in cellular models of AD compared to wild‐type controls, indicating a loss of mitochondrial membrane integrity and subsequent mtDNA leakage. Second, deficiency in aldehyde dehydrogenase 2 (ALDH2) exacerbates mtDNA damage and its cytosolic accumulation, leading to cGAS‐STING pathway activation and impaired mitophagy (Wang et al. 2020). Further mechanistic studies revealed that tau protein localizing to mitochondria can trigger mtDNA leakage and cGAS activation in microglia. This process inhibits the myocyte enhancer factor 2C (MEF2C)‐mediated transcriptional network in neurons, ultimately impairing cognitive function (Udeochu et al. 2023). Additionally, phospholipase D3 (PLD3) is a key lysosomal 5′–3′ exonuclease that primarily degrades mtDNA. In PLD3‐deficient cells, impaired lysosomal function leads to mtDNA leakage into the cytosol, activating the cGAS‐STING pathway and inducing the accumulation of APP‐CTF and cholesterol (Van Acker et al. 2023). In summary, the mtDNA‐cGAS‐STING signaling axis constitutes a crucial mechanism in the pathogenesis of AD.

### 4.3 Endoplasmic Reticulum Stress

The ER is the primary site for protein biosynthesis and processing, responsible for critical functions including the post‐translational modification, folding, and assembly of newly synthesized proteins (Nagar et al. 2023). The sustained accumulation of misfolded proteins induces ER stress, triggering the adaptive UPR to restore proteostasis by facilitating the degradation of aberrant proteins (Chen et al. 2023). In AD, the persistent accumulation of Aβ and p‐tau disrupts ER calcium homeostasis, impairs protein folding, and consequently induces profound ER stress. Studies have demonstrated that reducing pathological tau phosphorylation can alleviate ER stress‐mediated neurotoxicity (Park et al. 2009, Song et al. 2024). Furthermore, Aβ can increase intracellular calcium influx by modulating calcium channels, leading to disrupted cytoplasmic calcium homeostasis, ER stress, and subsequent memory impairment (Ghanbari‐Maman et al. 2019). Collectively, these findings establish ER stress as a critical contributor to AD pathogenesis.

A growing body of evidence indicates a robust interplay between ER stress and STING signaling activation. On one hand, ER stress can act as a potent inducer of STING signaling. For example, the ER stress inhibitor 4‐phenylbutyric acid (4‐PBA) has been shown to suppress STING‐IRF3 pathway activation (Li et al. 2023). Pharmacological studies indicate that UPR inducers can activate STING signaling and TBK1 phosphorylation, leading to IRF3 phosphorylation and its nuclear translocation (Liu et al. 2012). Furthermore, STING signaling activation is functionally linked to ER‐regulated Ca^2^⁺ homeostasis (Kwon et al. 2018). The stromal interaction molecule 1 (STIM1) binds to the calcium channel protein Orai1 to form a Ca^2^⁺ release‐activated Ca^2^⁺ (CRAC) channel, promoting extracellular Ca^2^⁺ influx that facilitates STING signaling activation (Srikanth et al. 2019). Conversely, activation of the cGAS‐STING signaling pathway can also induce ER stress. Studies confirm that cGAS‐STING activation exacerbates ER stress‐induced damage, while inhibition of cGAS with RU.521 alleviates ER stress (Huang et al. 2022). Moreover, cGAMP‐activated STING can interact with protein kinase R‐like ER kinase (PERK), a key UPR sensor that phosphorylates eukaryotic initiation factor 2α (eIF2α) to regulate ER homeostasis (Zhang et al. 2022, Wan et al. 2024). Critically, PERK activation promotes Aβ production and plaque deposition in AD models by upregulating the expression of activating transcription factor 4 (ATF4) and β‐site APP‐cleaving enzyme 1 (BACE1) (Hugon et al. 2017). Conversely, PERK knockout significantly ameliorates AD‐related pathology (Ma et al. 2013).

### 4.4 Nuclear DNA

In mammalian cells, genomic DNA is confined within the nuclear compartment. When cells die, nuclear DNA can be released into the extracellular space. Under normal physiological conditions, this extracellular DNA is efficiently removed by serum deoxyribonuclease I (DNase I) or, after phagocytic uptake, is broken down within lysosomes via DNase II, thus avoiding unintended immune stimulation (Kawane et al. 2001). Strong evidence indicates that self‐DNA released during cell death serves as a major trigger for the cGAS‐STING signaling pathway. For instance, in mouse models of myocardial infarction, widespread death of heart muscle cells liberates large amounts of self‐DNA, which in turn stimulates cGAS‐STING signaling, prompting interferon production and enhancing inflammation (King et al. 2017). In AD, multiple pathological drivers—such as Aβ deposition (Naderi et al. 2023), hyperphosphorylated tau (Thal and Tomé 2022), glutamate excitotoxicity (Wang and Reddy 2017), and neuroinflammation (Choi et al. 2023)—cause extensive neuronal loss in susceptible regions, including the hippocampus and cerebral cortex. As a result, nuclear DNA from dying neurons activates the cGAS‐STING pathway. Notably, brains of Alzheimer's patients contain thirty times more cells with fragmented DNA than those of healthy individuals (Lassmann et al. 1995), and this accumulation of damaged DNA strongly potentiates cGAS‐STING signaling and downstream molecular effectors. In senescent cells, reduced DNase expression leads to nuclear DNA buildup, provoking aberrant cGAS activation that drives the SASP via IFN‐β induction (Takahashi et al. 2018). Similarly, loss of DNase II function results in undegraded DNA escaping from lysosomes, which activates STING‐dependent signaling (Ahn et al. 2012). Relevant to AD, microglial deficiency in DNase II enhances cGAS‐STING activation, accelerates Aβ and tau pathology, and worsens cognitive impairment in mouse models (Li et al. 2025). Moreover, cGAS can detect nuclear DNA that enters the cytosol due to retrotransposon activity. Long Interspersed Nuclear Element‐1 (LINE‐1), for example, reverse transcribes its RNA into cDNA that activates the cGAS‐STING pathway. Inhibiting LINE‐1 reverse transcription lowers cytoplasmic DNA levels and thereby dampens cGAS‐STING activity (Gamdzyk et al. 2020). Cells also employ intrinsic mechanisms to restrain cGAS activation by self‐DNA. One such mechanism involves barrier‐to‐autointegration factor 1 (BAF), which sequesters nuclear DNA upon loss of nuclear envelope integrity, preventing cGAS recognition (Guey et al. 2020).

## 5 The Role of cGAS‐STING in AD

An imbalance between amyloid‐beta (Aβ) production and clearance is a cornerstone of AD pathology. Accumulating evidence demonstrates that the cGAS‐STING signaling pathway plays a significant role in regulating both the generation and clearance of Aβ. Clinical studies reveal a significant correlation between cerebrospinal fluid (CSF) IFN‐β levels and both P‐tau levels and the Aβ_42_/Aβ_40_ ratio in AD patients (Wang et al. 2024). The pathway influences Aβ dynamics through dual mechanisms. First, cGAS‐STING activation can promote Aβ production. Aβ is derived from the sequential proteolytic processing of APP. For instance, in cells with phospholipase D3 (PLD3) deficiency, lysosomal dysfunction leads to mtDNA accumulation and subsequent leakage into the cytosol. This cytosolic mtDNA activates STING signaling, increasing the production of APP‐CTF (Van Acker et al. 2023). Conversely, STING inhibition normalizes APP‐CTF levels. Furthermore, APP‐CTF accumulation itself induces excessive mitochondrial morphological changes and reactive oxygen species (ROS) accumulation, thereby driving mitochondrial dysfunction (Vaillant‐Beuchot et al. 2021). This dysfunction can cause mtDNA leakage into the cytosol, further activating cGAS signaling and creating a pathogenic feedback loop. Second, the cGAS‐STING pathway critically regulates the phagocytic function of microglia. As the resident phagocytes of the CNS, microglia clear Aβ aggregates and cellular debris under physiological conditions (Pluvinage et al. 2019). However, excessive Aβ phagocytosis can lead to mtDNA leakage into the cytosol, which activates cGAS signaling and exacerbates neuroinflammation. Yuan et al. (Yuan et al. 2025) demonstrated that inhibiting STING transcription—by targeting the G‐quadruplex structure in its promoter—downregulates STING expression, alleviates cellular senescence, and restores microglial capacity for Aβ phagocytosis. Supporting this, cGAS deficiency in microglia significantly reduces APP levels, impairs Aβ phagocytosis, and decreases plaque burden in the brains of 5xFAD mice (He et al. 2025). The pathway also intersects with key phagocytic regulators. Triggering receptor expressed on myeloid cells 2 (TREM2), a microglial surface receptor, is crucial for regulating phagocytic function. cGAMP promotes TREM2 expression through STING‐IRF3 signaling activation, thereby reducing Aβ deposition (Xu et al. 2019). Furthermore, cGAS deficiency in microglia inhibits the activation of neurotoxic A1‐type astrocytes, thereby mitigating Aβ‐induced neurotoxicity (Xie et al. 2023).

Neuroinflammation represents a fundamental mechanism underlying the pathogenesis of AD (Zhao 2024). Dysregulated activation of the cGAS‐STING signaling pathway—a central innate immune mechanism—is closely linked to both neuroinflammatory processes and AD progression (Yang et al. 2024). Increased expression of cGAS and STING has been detected predominantly in microglia from both AD patients and relevant mouse models (He et al. 2025). Supporting this observation, in the 5xFAD mouse model, p‐STING and IRF3 co‐localize with the microglial activation marker CD68 near Aβ plaques in the dentate gyrus, suggesting targeted cGAS‐STING pathway activation within microglia (Xie et al. 2023). These findings collectively demonstrate that the pro‐inflammatory influence of the cGAS‐STING pathway occurs mainly through microglial activity. Microglia, serving as the primary innate immune cells in the CNS, play a crucial role in AD‐related neuroinflammation. Experimental reduction of microglial cGAS or pharmacological inhibition of STING using H‐151 significantly alleviates neuroinflammatory responses in AD mouse models (Xie et al. 2023). The cGAS‐STING pathway plays a key role in mediating neuroinflammation associated with both Aβ and tau pathologies. In microglia, Aβ accumulation stimulates cGAS‐STING signaling, resulting in increased expression of interferon‐induced transmembrane protein 3 (IFITM3), polarization of microglia toward the M1 phenotype, and intensified neuroinflammatory responses (Wu et al. 2023). Likewise, tau interacts with polyglutamine‐binding protein 1 (PQBP1) to induce cGAS‐STING activation, which further stimulates microglial reactivity and augments neuroinflammation (Jin et al. 2021). Additionally, the NLRP3 inflammasome—highly expressed in microglia—contributes to inflammatory damage in AD (Heneka et al. 2013). Mitochondrial DNA, for instance, can activate the cGAS‐STING pathway in microglia, leading to NLRP3 inflammasome activation; this sequence is suppressed by the cGAS inhibitor RU.521 (Yang et al. 2024). Intriguingly, studies using the APP/PS1 mouse model reveal a more complex role: the STING agonist cGAMP paradoxically promotes a shift in microglial phenotype from proinflammatory M1 to protective M2, and upregulates TREM2, thereby reducing proinflammatory cytokine release (Xu et al. 2019). This anti‐inflammatory effect may be associated with enhanced phagocytic activity in microglia. Thus, a major therapeutic goal in AD is to harness the beneficial phagocytic functions of microglia while restraining their harmful inflammatory activation.

Autophagy is an evolutionarily conserved process in eukaryotic cells that depends on lysosomal activity to degrade cellular components, serving a central function in controlling protein metabolism and preserving cellular homeostasis. This system supports intracellular proteostasis by clearing damaged organelles, removing misfolded proteins, and recycling biological macromolecules (Liu et al. 2023). Impairments in autophagy are strongly associated with the development of AD (Zhang et al. 2021). Examinations of post‐mortem brain tissue from AD patients show a pronounced buildup of immature autophagosomes, along with reduced expression of critical autophagy‐related proteins, including LC3 and p62 (Zhang et al. 2023). Animal models provide additional evidence, indicating that suppression of Beclin‐1 increases the accumulation of APP, Aβ, and CTFs. In contrast, overexpression of Beclin‐1 boosts basal autophagic activity, which effectively reduces both Aβ aggregation and cognitive decline (Rocchi et al. 2017). The relationship between autophagy and the cGAS‐STING pathway is reciprocal. The cGAS‐STING signaling pathway is subject to regulatory control by autophagy. This regulation occurs, in part, through the autophagy‐initiating protein Beclin‐1, which binds directly to cGAS and inhibits its enzymatic activity, thereby suppressing the synthesis of cGAMP. This inhibition attenuates the production of type I interferons and, in conjunction with the autophagic degradation of cytoplasmic DNA, serves to prevent excessive or pathological cGAS activation (Liang et al. 2014). Mitophagy—the selective autophagic elimination of mitochondria—for instance, diminishes cytosolic mtDNA, thereby attenuating cGAS‐STING activation, particularly in aging (Jiménez‐Loygorri et al. 2024). On the other hand, cGAS‐STING signaling can also influence autophagic function. Cytosolic mtDNA leakage from compromised lysosomes activates the cGAS‐STING pathway, which may upregulate autophagic activity and unexpectedly result in the buildup of APP‐CTFs (Van Acker et al. 2023). Wang et al. demonstrated that melatonin effectively mitigates the APP/PS1‐induced suppression of mitochondrial autophagy (mitophagy) and impairment of cardiomyocyte function. Furthermore, they showed that pharmacological inhibition of the cGAS‐STING pathway abolished these beneficial effects of melatonin on mitophagy, cell survival, and cardiac function (Wang et al. 2020). Research by Lin et al. (Lin et al. 2021) demonstrated that inhibiting STING reduces autophagy through modulation of the mTOR pathway. Additionally, cGAS‐STING activation can disrupt the autophagy‐lysosome system, thereby promoting the accumulation and aggregation of tau protein (Zhang et al. 2025).

**FIGURE 3:**
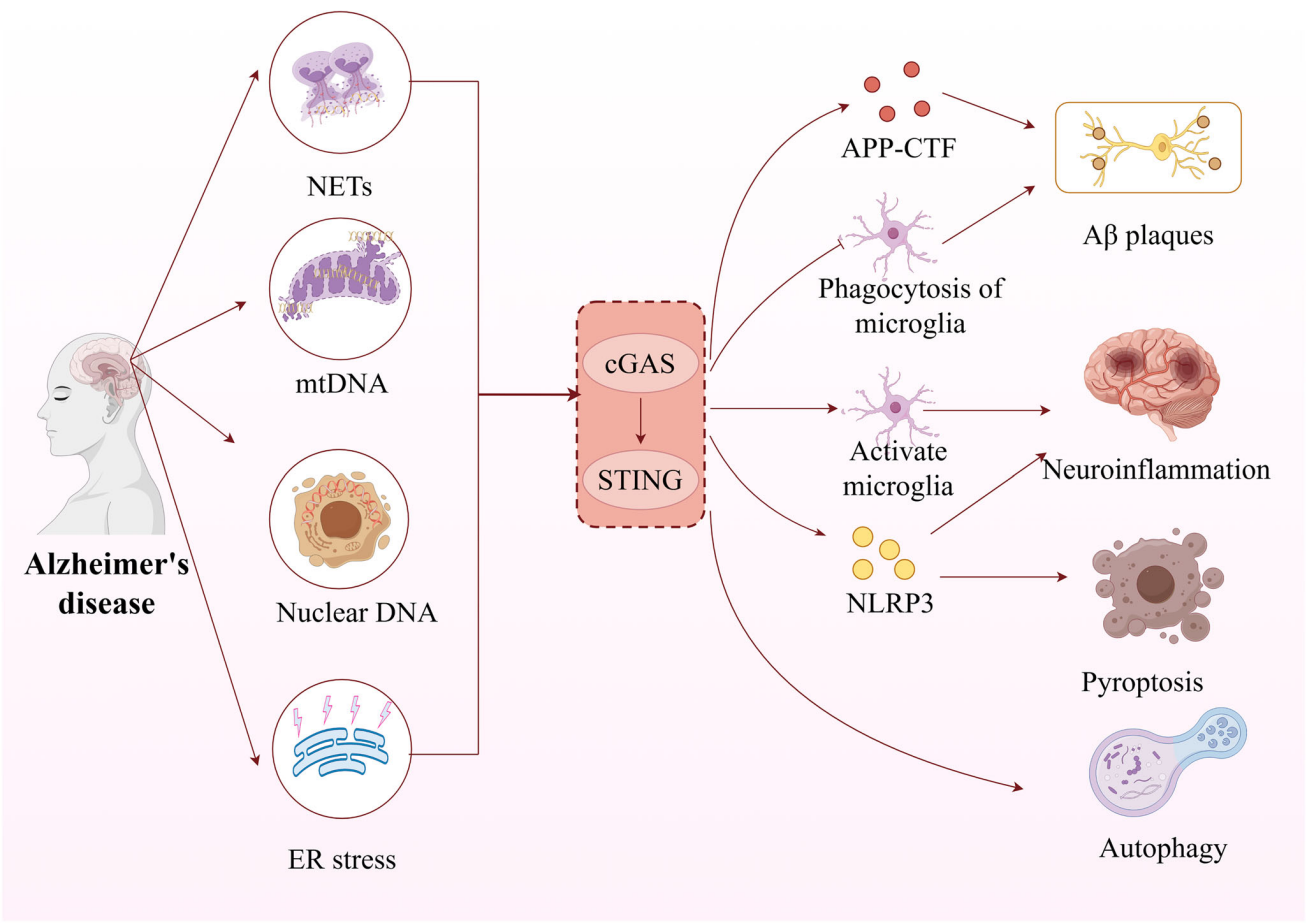
The relationship between the cGAS‐STING signaling pathway and the pathological mechanisms of AD.The role of the cGAS‐STING signaling pathway in AD pathology. AD leads to the production of neutrophil extracellular traps (NETs), endoplasmic reticulum (ER) stress, and the release of mitochondrial DNA (mtDNA) and nuclear DNA, which activate the cGAS‐STING signaling pathway, thereby exacerbating Aβ plaque deposition, neuroinflammation, pyroptosis, and autophagy (by Figdraw).

Pyroptosis, a highly inflammatory form of programmed cell death, is critically involved in the pathogenesis of AD (De Dios et al. 2023, Huang et al. 2022). This process is marked by the formation of plasma membrane pores mediated by GSDMD and functions as a key downstream component in canonical and non‐canonical inflammasome signaling (Man et al. 2017). Research using brain tissue from AD patients and animal models has shown that Aβ_1_₋_42_ triggers neuronal pyroptosis in the cortex, which correlates with increased levels of cleaved caspase‐1 (Han et al. 2020, Shen et al. 2021). Activation of the NLRP3 inflammasome serves as a primary mechanism driving pyroptosis in AD (Gaidt and Hornung 2018). Evidence indicates that both oligomeric and fibrillar Aβ species can stimulate the NLRP3 inflammasome, resulting in caspase‐1 activation and the generation of mature IL‐1β (Halle et al. 2008). The cGAS‐STING signaling pathway is closely associated with pyroptosis mediated by the NLRP3 inflammasome (Zhang et al. 2022). Specifically, stimulation of the cGAS‐STING pathway facilitates the assembly of the NLRP3 inflammasome in microglia, leading to subsequent pyroptotic cell death. In contrast, pharmacological blockade of cGAS using RU.521 or genetic silencing of STING attenuates NLRP3‐dependent pyroptosis in microglial cells (Ding et al. 2022). Collectively, these studies establish NLRP3 as a downstream effector of the cGAS‐STING signaling pathway. Evidence indicates that upon cytoplasmic DNA stimulation, STING activates the NLRP3 inflammasome through several distinct mechanisms. First, STING directly binds to NLRP3 within the endoplasmic reticulum, which promotes the NLRP3‐ASC interaction and facilitates ASC oligomerization, thereby driving inflammasome assembly (Ming et al. 2020). Second, through its TM5 domain, STING interacts with NLRP3 to remove its K48‐ and K63‐linked ubiquitin chains, a process that promotes NLRP3 activation (Wang et al. 2020). A third mechanism involves the transcriptional upregulation of NLRP3, whereby STING signaling enhances histone methylation at the NLRP3 promoter and recruits the transcription factor IRF3 to this region (Xiao et al. 2023). This mechanistic link is implicated in AD pathology; for instance, tooth loss has been shown to contribute to cognitive impairment in SAMP8 mice by promoting hippocampal mtDNA accumulation, upregulating the cGAS‐STING pathway, and subsequently triggering microglial pyroptosis. Additionally, manganese (Mn) exposure promotes both Aβ accumulation and tau hyperphosphorylation, which in turn stimulate the cGAS‐STING pathway and ultimately induce NLRP3 inflammasome activation (Liu et al. 2024) (Figure 3).

## 6 Treatment of the cGAS‐STING Pathway in AD

As highlighted in the preceding sections, the cGAS‐STING signaling pathway is a central driver of AD pathogenesis. Consequently, pharmacological modulation of this pathway holds significant promise as a novel therapeutic strategy for delaying AD progression. This review will now focus on targeted therapeutic strategies, particularly small‐molecule inhibitors and agonists, and will systematically summarize recent advances in modulating cGAS‐STING signaling in preclinical disease models.

### 6.1 cGAS Inhibitor

Inhibition of cGAS activity represents a core therapeutic strategy for suppressing the cGAS‐STING pathway. Developed cGAS inhibitors primarily fall into two mechanistic categories: those that act as competitive antagonists by binding directly to the enzyme's active site, and those that function allosterically by disrupting the interaction between cGAS and dsDNA (Zhou et al. 2023).

Early studies identified that antimalarial drugs, including hydroxychloroquine and quinacrine, can bind to dsDNA and prevent its interaction with cGAS (An et al. 2017). I The inhibitory oligodeoxynucleotide A151 suppresses cGAS catalytic activity by binding to its dsDNA‐binding domain, as demonstrated in THP‐1 human monocytes (Steinhagen et al. 2018). High‐throughput screening has further identified RU.365 and its benzothiazole analog RU.332 as compounds with significant inhibitory efficacy against murine cGAS (Vincent et al. 2017). Additionally, the acetyl‐donor aspirin can reduce cGAS enzymatic activity by promoting its acetylation (Dai et al. 2019).

In AD models, cGAS inhibitors have demonstrated efficacy. For instance, Udeochu et al. (Udeochu et al. 2023) reported that the small‐molecule inhibitors TDI‐6570 (a murine cGAS inhibitor) and TDI‐8246 (a human cGAS inhibitor) phenocopied the beneficial effects of cGAS knockout in both in vitro cultures and PS19 tauopathy mice. RU.521, an inhibitor that directly targets the cGAS catalytic site, blocks cGAMP synthesis, thereby inhibiting STING activation and downstream inflammatory cascades and ultimately alleviating chronic inflammation (Wiser et al. 2020, Shao et al. 2023). Animal studies have confirmed that RU.521 treatment reduces IFN‐β1 expression in mouse macrophages and significantly ameliorates Aβ pathology in 5xFAD mice (Xie et al. 2023). (Table 2)

**TABLE 2:** cGAS inhibitors.

Categories	Compound name	Pharmacological mechanisms	Reference
Antimalarial drugs	Hydroxychloroquine	Bind dsDNA to prevent its interaction with cGAS	(An et al. 2017)
	Quinacrine		
RU series compounds	RU.521	Competes with ATP and GTP for the active site of cGAS, decreasing cGAMP synthesis	(Vincent et al. 2017, Shao et al. 2023)
	RU.365		
	RU.332		
Aspirin		Maintains the acetylated inactive state of cGAS on Lys384, Lys394, or Lys414 and avoids attack by dsDNA	(Dai et al. 2019)
A151		dsDNA binding domain binding inhibits the catalytic activity of cGAS	(Steinhagen et al. 2018, Wu et al. 2023)

### 6.2 STING Inhibitor

As a core regulatory factor of innate immunity, STING is a promising therapeutic target, and its inhibitors have demonstrated significant potential for treating inflammatory diseases. To date, two primary strategies have been employed to develop STING inhibitors. The first strategy involves designing molecules that occupy the cyclic dinucleotide (CDN) binding site, acting as competitive antagonists. The second strategy focuses on identifying compounds that bind to cysteine residues (Cys88 or Cys91) near the transmembrane domain. These inhibitors disrupt STING palmitoylation, thereby reducing the recruitment of downstream signaling factors and preventing STING activation (Decout et al. 2021).

A major focus of STING inhibitor research targets its palmitoylation. Many compounds covalently bind to Cys88 or Cys91 in the transmembrane region to disrupt this essential post‐translational modification. Additionally, inhibitors targeting other sites on STING have also been identified. For example, the cyclic peptide Astin C, isolated from the aster plant, binds specifically to the CDN‐binding pocket in the STING C‐terminal domain. This binding prevents IRF3 recruitment while maintaining the STING‐TBK1 interaction, thereby inhibiting downstream innate immune responses (Li et al. 2018). Similarly, nitrofuran derivatives (e.g., compounds 20–23) inhibit STING palmitoylation and potently reduce STING‐mediated IFN‐β reporter activity (Haag et al. 2018). Notably, the antiviral drug remdesivir has been shown to inhibit STING expression, regulating lipid metabolism and inflammation in hepatocytes and alleviating non‐alcoholic fatty liver disease (NAFLD) in high‐fat diet models (Li and Su 2020).

The irreversible covalent inhibitor C‐176, which binds to the Cys91 site, significantly reduces serum type IFN‐I and IL‐6 levels induced by STING agonists (Li et al. 2024). In microglial cells treated with Aβ_25‐35_, C‐176—both as a monotherapy and in combination with the cGAS inhibitor RU.521—demonstrated potent anti‐neuroinflammatory effects (Haag et al. 2018, Wang et al. 2023). Mass spectrometry confirmed that H‐151 forms a covalent bond with Cys91, effectively inhibiting the type I interferon response, TBK1 phosphorylation, and human STING (hsSTING) palmitoylation. Both in vitro and in vivo studies have shown that H‐151 inhibits IFN‐β production and significantly suppresses the cytokine response to STING agonists (Haag et al. 2018). H‐151 treatment significantly ameliorates Aβ pathology in 5xFAD transgenic mice (Xie et al. 2023, Sharma et al. 2025). Furthermore, combination therapy with H‐151 and the soluble epoxide hydrolase inhibitor TPPU produces a synergistic anti‐AD effect, enhancing early‐stage Aβ phagocytosis and promoting late‐stage Aβ degradation (Fiala et al. 2025). Collectively, this research provides a strong rationale for the clinical translation of STING inhibitors. (Table 3)

**TABLE 3:** STING inhibitors.

Categories	Compound name	Pharmacological mechanisms	Reference
Astin C		Specifically binds to the C‐terminal activation pocket of STING, and prevents IRF3 recruitment and activation	(Li et al. 2018)
Nitrofuran derivatives	C‐176	Binds to the Cys91 residue on the STING protein, inhibiting its palmitoylation	(Kong et al. 2022, Li et al. 2024, Wang et al. 2023)
	C‐171		
	C‐178		
3‐acylaminoindole derivative	H‐151	Selective covalent antagonist of STING, reduce phosphorylation of TBK1 and suppress palmitoylation of human STING	(Xie et al. 2023, Haag et al. 2018)

### 6.3 Other Inhibitor

As a non‐canonical IKK family kinase, TBK1 interacts with the C‐terminal tail (CTT) of oligomerized STING. This association facilitates the phosphorylation of both STING and the transcription factor IRF3, initiating the production of IFN‐I and related cytokines (Zhang et al. 2019). Targeting TBK1 has therefore become a significant therapeutic approach for suppressing downstream STING signaling. The selective TBK1 inhibitor GSK8612 potently blocks Toll‐like receptor 3 (TLR3)‐mediated IRF3 phosphorylation in Ramos cells and reduces IFN‐I release in human primary monocytes. In monocyte‐derived macrophages, GSK8612 markedly attenuates the secretion of IFN‐β induced by dsDNA and cGAMP (Zeng et al. 2022).

In addition to synthetic compounds, a range of natural compounds and nanomaterials have shown promise in alleviating AD pathology through modulation of the cGAS‐STING pathway. For instance, nicotinamide adenine dinucleotide (NAD⁺) suppresses neuroinflammatory responses and cellular senescence by inhibiting cGAS‐STING signaling (Hou et al. 2021). Tetrahydroxystilbene glucoside (TSG) mediates neuroprotection via regulation of the cGAS‐STING pathway and suppression of NLRP3 inflammasome activation (Gao et al. 2023). Several natural compounds demonstrate therapeutic potential for AD by targeting the cGAS‐STING pathway. For instance, silymarin confers neuroprotection in sporadic AD models by mitigating iron‐induced injury and attenuating downstream STING‐mediated neuroinflammation (Liu et al. 2023). Similarly, icariin has been shown to inhibit glial activation and neuroinflammation via the cGAS‐STING pathway, thereby ameliorating cognitive deficits (Lu et al. 2025). Additionally, Platycodin D alleviates behavioral deficits, reduces Aβ accumulation, and mitigates mitochondrial damage in an AD model by suppressing cGAS‐STING signaling (Saida et al. 2025). Zhang et al. (2025) developed a nanozyme based on a covalent organic framework (COF) responsive to copper ions, which selectively targets and inhibits cGAS‐STING signaling in the AD brain, presenting an innovative therapeutic approach. While no cGAS‐STING‐targeting drugs are currently approved for AD treatment, this growing body of research provides a crucial foundation for the development of novel disease‐modifying therapies.

## 7 Conclusions

Significant advances have been made in understanding the cGAS‐STING signaling pathway in recent years. Extensive evidence now underscores its pivotal regulatory role in the CNS, linking its dysregulation to neuroinflammation in neurodegenerative diseases such as AD. Notably, the pathway's function is not exclusively pro‐inflammatory but exhibits a context‐dependent duality. For instance, the STING agonist cGAMP has been shown to improve cognitive function in AD mouse models and promote the polarization of glial cells toward an anti‐inflammatory M2 phenotype (Xu et al. 2019). This finding contrasts with the perception of cGAS‐STING as a purely detrimental pathway. This paradox suggests that the pathway's effects are modulated by factors such as activation intensity, duration, cellular context, and disease stage; moderate acute activation may support immune surveillance and repair, whereas chronic excessive activation drives a self‐perpetuating cycle of neuroinflammation and injury. Functioning as a central node in the AD pathological network, the cGAS‐STING pathway integrates signals from mitochondrial dysfunction, endoplasmic reticulum stress, cell death, and inflammatory responses to accelerate disease progression. Consequently, the development of selective and effective modulators of this pathway holds considerable therapeutic promise. Small‐molecule inhibitors and other compounds targeting this pathway have demonstrated efficacy in preclinical models, providing a rationale for developing disease‐modifying AD therapies. Future research must elucidate the cell‐type and stage‐specific mechanisms of this pathway and explore multi‐target therapeutic strategies. Such strategies could include combining STING inhibitors with Aβ/tau‐targeting drugs or developing dual‐functional molecules that concurrently suppress neuroinflammation and promote autophagy, enabling a more comprehensive blockade of pathology and enhanced cognitive protection. Furthermore, delineating the dynamic changes and dual roles of the cGAS‐STING pathway across the AD continuum, from preclinical to dementia stages, will be critical for founding personalized immunomodulatory therapies.

## Author Contributions

Xue Li: writing – original draft, writing – review and editing. Wei Gao：writing – review and editing, writing – original draft. Qiuyan Ye: writing – review and editing, writing – original draft. Honglin Li: conceptualization, writing – review and editing, funding acquisition.All authors contributed to the study conception and design. Honglin Li: conceptualization. Xue Li: drafting and revising the manuscript text. Wei Gao: drawing figure and revising the manuscript text. Qiuyan Ye: drawing figure.

## Funding

This study was supported by the National Natural Science Foundation of China (82105035) and Natural Science Foundation of Heilongjiang Province of China (LH2023H064) awarded to Honglin Li.

## Ethics Statement

The authors have nothing to report.

## Data Availability

Data availability is not applicable to this article as no new data were created or analyzed in this study.

## References

[brb371130-bib-0001] Gui, X. , H. Yang , T. Li , et al. 2019. “Autophagy Induction via STING Trafficking Is a Primordial Function of the cGAS Pathway.” Nature 567, no. 7747: 262–266. 10.1038/s41586-019-1006-9. 30842662 PMC9417302

[brb371130-bib-0002] Ablasser, A. , M. Goldeck , T. Cavlar , et al. 2013. “cGAS Produces a 2'‐5'‐Linked Cyclic Dinucleotide Second Messenger That Activates STING.” Nature 498, no. 7454: 380–384. 10.1038/nature12306. 23722158 PMC4143541

[brb371130-bib-0003] Ahn, J. , D. Gutman , S. Saijo , and G. N. Barber . 2012. “STING Manifests Self DNA‐Dependent Inflammatory Disease.” PNAS 109, no. 47: 19386–19391. 10.1073/pnas.1215006109. 23132945 PMC3511090

[brb371130-bib-0004] Ahtiluoto, S. , T. Polvikoski , M. Peltonen , et al. 2010. “Diabetes, Alzheimer Disease, and Vascular Dementia: A Population‐Based Neuropathologic Study.” Neurology 75, no. 13: 1195–1202. 10.1212/WNL.0b013e3181f4d7f8. 20739645

[brb371130-bib-0005] An, J. , M. Minie , T. Sasaki , J. J. Woodward , and K. B. Elkon . 2017. “Antimalarial Drugs as Immune Modulators: New Mechanisms for Old Drugs.” Annual Review of Medicine 68: 317–330. 10.1146/annurev-med-043015-123453. 27813878

[brb371130-bib-0006] Bai, J. , C. Cervantes , J. Liu , et al. 2017. “DsbA‐L Prevents Obesity‐Induced Inflammation and Insulin Resistance by Suppressing the mtDNA Release‐Activated cGAS‐cGAMP‐STING Pathway.” PNAS 114, no. 46: 12196–12201. 10.1073/pnas.1708744114. 29087318 PMC5699051

[brb371130-bib-0007] Biessels, G. J. , S. Staekenborg , E. Brunner , C. Brayne , and P. Scheltens . 2006. “Risk of Dementia in Diabetes Mellitus: A Systematic Review.” Lancet Neurology 5, no. 1: 64–74. 10.1016/s1474-4422(05)70284-2. 16361024

[brb371130-bib-0008] Chauhan, C. , and R. K. Kaundal . 2023. “The Role of cGAS‐STING Signaling in Ischemic Stroke: From Immune Response to Therapeutic Targeting.” Drug Discovery Today 28, no. 11: 103792. 10.1016/j.drudis.2023.103792. 37783431

[brb371130-bib-0009] Chen, X. , C. Shi , M. He , S. Xiong , and X. Xia . 2023. “Endoplasmic Reticulum Stress: Molecular Mechanism and Therapeutic Targets.” Signal Transduction and Targeted Therapy 8, no. 1: 352. 10.1038/s41392-023-01570-w. 37709773 PMC10502142

[brb371130-bib-0010] Choi, S. B. , S. Kwon , J. H. Kim , N. H. Ahn , J. H. Lee , and S. H. Yang . 2023. “The Molecular Mechanisms of Neuroinflammation in Alzheimer's Disease, the Consequence of Neural Cell Death.” International Journal of Molecular Sciences 24, no. 14: 11757. 10.3390/ijms241411757. 37511515 PMC10380735

[brb371130-bib-0011] Cole, J. B. , and J. C. Florez . 2020. “Genetics of Diabetes Mellitus and Diabetes Complications.” Nature Reviews Nephrology 16, no. 7: 377–390. 10.1038/s41581-020-0278-5. 32398868 PMC9639302

[brb371130-bib-0012] Cruz, V. H. , E. N. Arner , K. W. Wynne , P. E. Scherer , and R. A. Brekken . 2018. “Loss of Tbk1 Kinase Activity Protects Mice From Diet‐induced Metabolic Dysfunction.” Molecular Metabolism 16: 139–149. 10.1016/j.molmet.2018.06.007. 29935921 PMC6157474

[brb371130-bib-0013] Dai, J. , Y.‐J. Huang , X. He , et al. 2019. “Acetylation Blocks cGAS Activity and Inhibits Self‐DNA‐Induced Autoimmunity.” Cell 176, no. 6: 1447–1460.e14. 10.1016/j.cell.2019.01.016. 30799039 PMC8274936

[brb371130-bib-0014] De Dios, C. , X. Abadin , V. Roca‐Agujetas , et al. 2023. “Inflammasome Activation Under High Cholesterol Load Triggers a Protective Microglial Phenotype While Promoting Neuronal Pyroptosis.” Translational Neurodegeneration 12, no. 1: 10. 10.1186/s40035-023-00343-3. 36895045 PMC9996936

[brb371130-bib-0015] Decout, A. , J. D. Katz , S. Venkatraman , and A. Ablasser . 2021. “The cGAS‐STING Pathway as a Therapeutic Target in Inflammatory Diseases.” Nature Reviews Immunology 21, no. 9: 548–569. 10.1038/s41577-021-00524-z. PMC8029610 33833439

[brb371130-bib-0016] Ding, R. , H. Li , Y. Liu , et al. 2022. “Activating cGAS‐STING Axis Contributes to Neuroinflammation in CVST Mouse Model and Induces Inflammasome Activation and Microglia Pyroptosis.” Journal of Neuroinflammation 19, no. 1: 137. 10.1186/s12974-022-02511-0. 35689216 PMC9188164

[brb371130-bib-0017] Dintica, C. S. , and K. Yaffe . 2022. “Epidemiology and Risk Factors for Dementia.” The Psychiatric Clinics of North America 45, no. 4: 677–689. 10.1016/j.psc.2022.07.011. 36396272

[brb371130-bib-0018] Doruk, H. , M. I. Naharci , E. Bozoglu , A. T. Isik , and S. Kilic . 2010. “The Relationship Between Body Mass Index and Incidental Mild Cognitive Impairment, Alzheimer's Disease and Vascular Dementia in Elderly.” Journal of Nutrition, Health and Aging 14, no. 10: 834–838. 10.1007/s12603-010-0113-y.21125201

[brb371130-bib-0019] Dou, Z. , K. Ghosh , M. G. Vizioli , et al. 2017. “Cytoplasmic Chromatin Triggers Inflammation in Senescence and Cancer.” Nature 550, no. 7676: 402–406. 10.1038/nature24050. 28976970 PMC5850938

[brb371130-bib-0020] Fiala, M. , B. D. Hammock , S. H. Hwang , et al. 2025. “Inhibitors of Soluble Epoxide Hydrolase and cGAS/STING Repair Defects in Amyloid‐β Clearance Underlying Vascular Complications of Alzheimer's Disease.” Journal of Alzheimer's Disease 104, no. 1: 150–157. 10.1177/13872877241305965. PMC11969680 39962970

[brb371130-bib-0021] Gaidt, M. M. , and V. Hornung . 2018. “The NLRP3 Inflammasome Renders Cell Death Pro‐Inflammatory.” Journal of Molecular Biology 430, no. 2: 133–141. 10.1016/j.jmb.2017.11.013. 29203171

[brb371130-bib-0022] Gamdzyk, M. , D. M. Doycheva , C. Araujo , et al. 2020. “cGAS/STING Pathway Activation Contributes to Delayed Neurodegeneration in Neonatal Hypoxia‐Ischemia Rat Model: Possible Involvement of LINE‐1.” Molecular Neurobiology 57, no. 6: 2600–2619. 10.1007/s12035-020-01904-7. 32253733 PMC7260114

[brb371130-bib-0023] Gao, D. , J.‐P. Hao , B‐Y. Li, et al. 2023. “Tetrahydroxy Stilbene Glycoside Ameliorates Neuroinflammation for Alzheimer's Disease via cGAS‐STING.” European Journal of Pharmacology 953: 175809. 10.1016/j.ejphar.2023.175809. 37328043

[brb371130-bib-0024] Ghanbari‐Maman, A. , F. Ghasemian‐Roudsari , S. Aliakbari , et al. 2019. “Calcium Channel Blockade Ameliorates Endoplasmic Reticulum Stress in the Hippocampus Induced by Amyloidopathy in the Entorhinal Cortex.” Iranian Journal of Pharmaceutical Research 18, no. 3: 1466–1476. 10.22037/ijpr.2019.111532.13216. 32641955 PMC6934951

[brb371130-bib-0025] Guey, B. , M. Wischnewski , A. Decout , et al. 2020. “BAF Restricts cGAS on Nuclear DNA to Prevent Innate Immune Activation.” Science 369, no. 6505: 823–828. 10.1126/science.aaw6421. 32792394

[brb371130-bib-0026] Gulen, M. F. , N. Samson , A. Keller , et al. 2023. “cGAS‐STING Drives Ageing‐Related Inflammation and Neurodegeneration.” Nature 620, no. 7973: 374–380. 10.1038/s41586-023-06373-1. 37532932 PMC10412454

[brb371130-bib-0027] Haag, S. M. , M. F. Gulen , L. Reymond , et al. 2018. “Targeting STING With Covalent Small‐Molecule Inhibitors.” Nature 559, no. 7713: 269–273. 10.1038/s41586-018-0287-8. 29973723

[brb371130-bib-0028] Halle, A. , V. Hornung , G. C. Petzold , et al. 2008. “The NALP3 Inflammasome Is Involved in the Innate Immune Response to Amyloid‐Beta.” Nature Immunology 9, no. 8: 857–865. 10.1038/ni.1636. 18604209 PMC3101478

[brb371130-bib-0029] Ham, H. J. , S.‐B. Han , J. Yun , et al. 2019. “Bee Venom Phospholipase A2 Ameliorates Amyloidogenesis and Neuroinflammation Through Inhibition of Signal Transducer and Activator of Transcription‐3 Pathway in Tg2576 Mice.” Translational Neurodegeneration 8: 26. 10.1186/s40035-019-0167-7. 31592103 PMC6774221

[brb371130-bib-0030] Han, C. , Y. Yang , Q. Guan , et al. 2020. “New Mechanism of Nerve Injury in Alzheimer's Disease: β‐amyloid‐induced Neuronal Pyroptosis.” Journal of Cellular and Molecular Medicine 24, no. 14: 8078–8090. 10.1111/jcmm.15439. 32521573 PMC7348172

[brb371130-bib-0031] He, S. , X. Li , N. Mittra , et al. 2025. “Microglial cGAS Deletion Preserves Intercellular Communication and Alleviates Amyloid‐β‐Induced Pathogenesis of Alzheimer's Disease.” Advanced Science 12, no. 12: e2410910. 10.1002/advs.202410910. 39908354 PMC11948024

[brb371130-bib-0032] Heneka, M. T. , M. P. Kummer , A. Stutz , et al. 2013. “NLRP3 is Activated in Alzheimer's Disease and Contributes to Pathology in APP/PS1 Mice.” Nature 493, no. 7434: 674–678. 10.1038/nature11729. 23254930 PMC3812809

[brb371130-bib-0033] Hou, Y. , X. Dan , M. Babbar , et al. 2019. “Ageing as a Risk Factor for Neurodegenerative Disease.” Nature Reviews Neurology 15, no. 10: 565–581. 10.1038/s41582-019-0244-7. 31501588

[brb371130-bib-0034] Hou, Y. , Y. Wei , S. Lautrup , et al. 2021. “NAD(+) Supplementation Reduces Neuroinflammation and Cell Senescence in a Transgenic Mouse Model of Alzheimer's Disease via cGAS‐STING.” PNAS 118, no. 37: e2011226118. 10.1073/pnas.2011226118. 34497121 PMC8449423

[brb371130-bib-0035] Huang, F.‐Q. , H.‐F. Wang , T. Yang , et al. 2025. “Ceramides Increase Mitochondrial Permeabilization to Trigger mtDNA‐Dependent Inflammation in Astrocytes During Brain Ischemia.” Metabolism: Clinical and Experimental 166: 156161. 10.1016/j.metabol.2025.156161. 39956315

[brb371130-bib-0036] Huang, R. , Q. Shi , S. Zhang , et al. 2022. “Inhibition of the cGAS‐STING Pathway Attenuates Lung Ischemia/Reperfusion Injury via Regulating Endoplasmic Reticulum Stress in Alveolar Epithelial Type II Cells of Rats.” Journal of Inflammation Research 15: 5103–5119. 10.2147/jir.S365970. 36091334 PMC9462969

[brb371130-bib-0037] Huang, Y. , X. Li , G. Luo , et al. 2022. “Pyroptosis as a Candidate Therapeutic Target for Alzheimer's Disease.” Frontiers in Aging Neuroscience 14: 996646. 10.3389/fnagi.2022.996646. 36185484 PMC9520296

[brb371130-bib-0038] Hugon, J. , F. Mouton‐Liger , J. Dumurgier , and C. Paquet . 2017. “PKR Involvement in Alzheimer's Disease.” Alzheimer's Research & Therapy 9, no. 1: 83. 10.1186/s13195-017-0308-0. PMC5629792 28982375

[brb371130-bib-0039] Jauhari, A. , S. V. Baranov , Y. Suofu , et al. 2020. “Melatonin Inhibits Cytosolic Mitochondrial DNA‐Induced Neuroinflammatory Signaling in Accelerated Aging and Neurodegeneration.” Journal of Clinical Investigation 130, no. 6: 3124–3136. 10.1172/jci135026. 32182222 PMC7260019

[brb371130-bib-0040] Jia, Y. , F. Li , Z. Liu , et al. 2024. “Interaction Between the SFTSV Envelope Glycoprotein Gn and STING Inhibits the Formation of the STING‐TBK1 Complex and Suppresses the NF‐κB Signaling Pathway.” Journal of Virology 98, no. 3: e0181523. 10.1128/jvi.01815-23. 38421179 PMC10949458

[brb371130-bib-0041] Jiménez‐Loygorri, J. I. , B. Villarejo‐Zori , Á. Viedma‐Poyatos , et al. 2024. “Mitophagy Curtails Cytosolic mtDNA‐Dependent Activation of cGAS/STING Inflammation During Aging.” Nature Communications 15, no. 1: 830. 10.1038/s41467-024-45044-1. PMC10821893 38280852

[brb371130-bib-0042] Jin, M. , H. Shiwaku , H. Tanaka , et al. 2021. “Tau Activates Microglia via the PQBP1‐cGAS‐STING Pathway to Promote Brain Inflammation.” Nature Communications 12, no. 1: 6565. 10.1038/s41467-021-26851-2. PMC8592984 34782623

[brb371130-bib-0043] Joshi, R. , V. Brezani , G. M. Mey , et al. 2024. “IRF3 Regulates Neuroinflammatory Responses and the Expression of Genes Associated With Alzheimer's Disease.” Journal of Neuroinflammation 21, no. 1: 212. 10.1186/s12974-024-03203-7. 39215356 PMC11363437

[brb371130-bib-0044] Kawane, K. , H. Fukuyama , G. Kondoh , et al. 2001. “Requirement of DNase II for Definitive Erythropoiesis in the Mouse Fetal Liver.” Science 292, no. 5521: 1546–1549. 10.1126/science.292.5521.1546. 11375492

[brb371130-bib-0045] Kim, J. , H. S. Kim , and J. H. Chung . 2023. “Molecular Mechanisms of Mitochondrial DNA Release and Activation of the cGAS‐STING Pathway.” Experimental & Molecular Medicine 55, no. 3: 510–519. 10.1038/s12276-023-00965-7. 36964253 PMC10037406

[brb371130-bib-0046] King, K. R. , A. D. Aguirre , Y.‐X. Ye , et al. 2017. “IRF3 and Type I Interferons Fuel a Fatal Response to Myocardial Infarction.” Nature Medicine 23, no. 12: 1481–1487. 10.1038/nm.4428. PMC6477926 29106401

[brb371130-bib-0047] Kong, F. , X. Jiang , R. Wang , S. Zhai , Y. Zhang , and D. Wang . 2020. “Forsythoside B Attenuates Memory Impairment and Neuroinflammation via Inhibition on NF‐κB Signaling in Alzheimer's Disease.” Journal of Neuroinflammation 17, no. 1: 305. 10.1186/s12974-020-01967-2. 33059746 PMC7565774

[brb371130-bib-0048] Kong, L. , W. Li , E. Chang , et al. 2022. “mtDNA‐STING Axis Mediates Microglial Polarization via IRF3/NF‐κB Signaling After Ischemic Stroke.” Frontiers in Immunology 13: 860977. 10.3389/fimmu.2022.860977. 35450066 PMC9017276

[brb371130-bib-0049] Kretzschmar, G. C. , V. Bumiller‐Bini , M. A. Gasparetto Filho , et al. 2021. “Neutrophil Extracellular Traps: A Perspective of Neuroinflammation and Complement Activation in Alzheimer's Disease.” Frontiers in Molecular Biosciences 8: 630869. 10.3389/fmolb.2021.630869. 33898514 PMC8060499

[brb371130-bib-0050] Kumari, R. , and P. Jat . 2021. “Mechanisms of Cellular Senescence: Cell Cycle Arrest and Senescence Associated Secretory Phenotype.” Frontiers in Cell and Developmental Biology 9: 645593. 10.3389/fcell.2021.645593. 33855023 PMC8039141

[brb371130-bib-0051] Kwon, D. , H. Sesaki , and S. J. Kang . 2018. “Intracellular Calcium Is a Rheostat for the STING Signaling Pathway.” Biochemical and Biophysical Research Communications 500, no. 2: 497–503. 10.1016/j.bbrc.2018.04.117. 29673589

[brb371130-bib-0052] Lassmann, H. , C. Bancher , H. Breitschopf , et al. 1995. “Cell Death in Alzheimer's Disease Evaluated by DNA Fragmentation in Situ.” Acta Neuropathologica 89, no. 1: 35–41. 10.1007/bf00294257. 7709729

[brb371130-bib-0053] Lee, E. B. 2011. “Obesity, Leptin, and Alzheimer's Disease.” Annals of the New York Academy of Sciences 1243: 15–29. 10.1111/j.1749-6632.2011.06274.x. 22211890 PMC3564488

[brb371130-bib-0054] Lei, Y. , J. J. Vanportfliet , Y.‐F. Chen , et al. 2023. “Cooperative Sensing of Mitochondrial DNA by ZBP1 and cGAS Promotes Cardiotoxicity.” Cell 186, no. 14: 3013–3032.e22. 10.1016/j.cell.2023.05.039. 37352855 PMC10330843

[brb371130-bib-0055] Li, L.‐J. , S.‐Y. Liang , X.‐Y. Sun , et al. 2025. “Microglial Double Stranded DNA Accumulation Induced by DNase II Deficiency Drives Neuroinflammation and Neurodegeneration.” Journal of Neuroinflammation 22, no. 1: 11. 10.1186/s12974-025-03333-6. 39833906 PMC11745000

[brb371130-bib-0056] Li, Q. , P. Wu , Q. Du , U. Hanif , H. Hu , and K. Li . 2024. “cGAS‐STING, an Important Signaling Pathway in Diseases and Their Therapy.” MedComm (2020) 5, no. 4: e511. 10.1002/mco2.511. 38525112 PMC10960729

[brb371130-bib-0057] Li, S. , Z. Hong , Z. Wang , et al. 2018. “The Cyclopeptide Astin C Specifically Inhibits the Innate Immune CDN Sensor STING.” Cell Reports 25, no. 12: 3405–3421.e7. 10.1016/j.celrep.2018.11.097. 30566866

[brb371130-bib-0058] Li, Y. N. , and Y. Su . 2020. “Remdesivir Attenuates High Fat Diet (HFD)‐Induced NAFLD by Regulating Hepatocyte Dyslipidemia and Inflammation via the Suppression of STING.” Biochemical and Biophysical Research Communications 526, no. 2: 381–388. 10.1016/j.bbrc.2020.03.034. 32223926 PMC7194706

[brb371130-bib-0059] Li, Y. , H. Chen , Q. Yang, et al. 2022. “Increased Drp1 Promotes Autophagy and ESCC Progression by mtDNA Stress Mediated cGAS‐STING Pathway.” Journal of Experimental & Clinical Cancer Research 41, no. 1: 76. 10.1186/s13046-022-02262-z. 35209954 PMC8867650

[brb371130-bib-0060] Li, Y. , J. Cui , L. Liu , et al. 2024. “mtDNA Release Promotes cGAS‐STING Activation and Accelerated Aging of Postmitotic Muscle Cells.” Cell Death & Disease 15, no. 7: 523. 10.1038/s41419-024-06863-8. 39039044 PMC11263593

[brb371130-bib-0061] Li, Y. , H. Lin , H. Tang , et al. 2023. “The STING‐IRF3 Signaling Pathway, Mediated by Endoplasmic Reticulum Stress, Contributes to Impaired Myocardial Autophagic Flux After Ischemia/Reperfusion.” Journal of Cardiovascular Pharmacology 82, no. 5: 389–399. 10.1097/fjc.0000000000001465. 37851150

[brb371130-bib-0062] Liang, Q. , G. J. Seo , Y. J. Choi , et al. 2014. “Crosstalk Between the cGAS DNA Sensor and Beclin‐1 Autophagy Protein Shapes Innate Antimicrobial Immune Responses.” Cell Host & Microbe 15, no. 2: 228–238. 10.1016/j.chom.2014.01.009. 24528868 PMC3950946

[brb371130-bib-0063] Lin, F. , X. Yao , C. Kong , et al. 2021. “25‐Hydroxycholesterol Protecting From Cerebral Ischemia‐Reperfusion Injury Through the Inhibition of STING Activity.” Aging 13, no. 16: 20149–20163. 10.18632/aging.203337. 34406977 PMC8436919

[brb371130-bib-0064] Lin, S.‐P. , J.‐X. Wei , J.‐S. Hu , et al. 2021. “Artemisinin Improves Neurocognitive Deficits Associated With Sepsis by Activating the AMPK Axis in Microglia.” Acta Pharmacologica Sinica 42, no. 7: 1069–1079. 10.1038/s41401-021-00634-3. 33758353 PMC8209200

[brb371130-bib-0065] Liu, D. , H. Wu , C. Wang , et al. 2019. “STING Directly Activates Autophagy to Tune the Innate Immune Response.” Cell Death and Differentiation 26, no. 9: 1735–1749. 10.1038/s41418-018-0251-z. 30568238 PMC6748081

[brb371130-bib-0066] Liu, H. , W.‐L. Cheng , X. Jiang, et al. 2017. “Ablation of Interferon Regulatory Factor 3 Protects Against Atherosclerosis in Apolipoprotein E‐Deficient Mice.” Hypertension 69, no. 3: 510–520. 10.1161/hypertensionaha.116.08395. 28115514

[brb371130-bib-0067] Liu, J. , Z. Zhang , S. Zhong , et al. 2024. “Fecal Microbiome Transplantation Alleviates Manganese‐Induced Neurotoxicity by Altering the Composition and Function of the Gut Microbiota via the cGAS‐STING/NLRP3 Pathway.” Science of the Total Environment 951: 175681. 10.1016/j.scitotenv.2024.175681. 39173756

[brb371130-bib-0068] Liu, N. , X. Pang , H. Zhang , and P. Ji,. 2021. “The cGAS‐STING Pathway in Bacterial Infection and Bacterial Immunity.” Frontiers in Immunology 12: 814709. 10.3389/fimmu.2021.814709. 35095914 PMC8793285

[brb371130-bib-0069] Liu, P. , W. Chen , Y.u Kang, et al. 2023. “Silibinin Ameliorates STING‐Mediated Neuroinflammation via Downregulation of Ferroptotic Damage in a Sporadic Alzheimer's Disease Model.” Archives of Biochemistry and Biophysics 744: 109691. 10.1016/j.abb.2023.109691. 37473980

[brb371130-bib-0070] Liu, R. M. 2022. “Aging, Cellular Senescence, and Alzheimer's Disease.” International Journal of Molecular Sciences 23, no. 4: 1989. 10.3390/ijms23041989. 35216123 PMC8874507

[brb371130-bib-0071] Liu, S. , S. Yao , H. Yang , S. Liu , and Y. Wang . 2023. “Autophagy: Regulator of Cell Death.” Cell Death & Disease 14, no. 10: 648. 10.1038/s41419-023-06154-8. 37794028 PMC10551038

[brb371130-bib-0072] Liu, Y.‐P. , L. Zeng , A. Tian , et al. 2012. “Endoplasmic Reticulum Stress Regulates the Innate Immunity Critical Transcription Factor IRF3.” Journal of Immunology 189, no. 9: 4630–4639. 10.4049/jimmunol.1102737. PMC3478468 23028052

[brb371130-bib-0073] Long, Z. J. , J. D. Wang , J. Q. Xu , X. X. Lei , and Q. Liu . 2022. “cGAS/STING Cross‐Talks With Cell Cycle and Potentiates Cancer Immunotherapy.” Molecular Therapy 30, no. 3: 1006–1017. 10.1016/j.ymthe.2022.01.044. 35121107 PMC8899703

[brb371130-bib-0074] Lu, F. , L. Li , B. Zheng , et al. 2025. “Icariin Alleviates Cognitive Dysfunction by Reducing Neuroinflammation via the cGAS‐STING Pathway.” Journal of Ethnopharmacology 350: 120010. 10.1016/j.jep.2025.120010. 40403897

[brb371130-bib-0075] Lv, J. , X. Zhu , C. Xing , et al. 2023. “Stimulator of Interferon Genes (STING): Key Therapeutic Targets in Ischemia/Reperfusion Injury.” Biomedicine & Pharmacotherapy 167: 115458. 10.1016/j.biopha.2023.115458. 37699319

[brb371130-bib-0076] Ma, T. , M. A. Trinh , A. J. Wexler , et al. 2013. “Suppression of eIF2α Kinases Alleviates Alzheimer's Disease‐Related Plasticity and Memory Deficits.” Nature Neuroscience 16, no. 9: 1299–1305. 10.1038/nn.3486. 23933749 PMC3756900

[brb371130-bib-0077] Man, S. M. , R. Karki , and T. D. Kanneganti . 2017. “Molecular Mechanisms and Functions of Pyroptosis, Inflammatory Caspases and Inflammasomes in Infectious Diseases.” Immunological Reviews 277, no. 1: 61–75. 10.1111/imr.12534. 28462526 PMC5416822

[brb371130-bib-0078] Ming, S.‐L.i , L. Zeng , Y.u‐K. Guo , et al. 2020. “The Human‐Specific STING Agonist G10 Activates Type I Interferon and the NLRP3 Inflammasome in Porcine Cells.” Frontiers in Immunology 11: 575818. 10.3389/fimmu.2020.575818. 33072119 PMC7543045

[brb371130-bib-0079] Moser, V. A. , and C. J. Pike . 2016. “Obesity and Sex Interact in the Regulation of Alzheimer's Disease.” Neuroscience & Biobehavioral Reviews 67: 102–118. 10.1016/j.neubiorev.2015.08.021. 26708713 PMC4912955

[brb371130-bib-0080] Naderi, S. , F. Khodagholi , H. G. Pourbadie , et al. 2023. “Role of Amyloid Beta (25‐35) Neurotoxicity in the Ferroptosis and Necroptosis as Modalities of Regulated Cell Death in Alzheimer's Disease.” Neurotoxicology 94: 71–86. 10.1016/j.neuro.2022.11.003. 36347329

[brb371130-bib-0081] Nagar, P. , P. Sharma , R. Dhapola , S. Kumari , B. Medhi , and D. HariKrishnaReddy . 2023. “Endoplasmic Reticulum Stress in Alzheimer's Disease: Molecular Mechanisms and Therapeutic Prospects.” Life Sciences 330: 121983. 10.1016/j.lfs.2023.121983. 37524162

[brb371130-bib-0082] Nichols, E. , J. D. Steinmetz , S. E. Vollset , et al. 2021. “Estimation of the Global Prevalence of Dementia in 2019 and Forecasted Prevalence in 2050: An Analysis for the Global Burden of Disease Study 2019.” The Lancet Public Health 7, no. 2: e105–e125. 10.1016/s2468-2667(21)00249-8. PMC8810394 34998485

[brb371130-bib-0083] Park, Y. J. , Y. M. Jang , and Y. H. Kwon . 2009. “Isoflavones Prevent Endoplasmic Reticulum Stress‐Mediated Neuronal Degeneration by Inhibiting Tau Hyperphosphorylation in SH‐SY5Y Cells.” Journal of Medicinal Food 12, no. 3: 528–535. 10.1089/jmf.2008.1069. 19627200

[brb371130-bib-0084] Passarella, S. , S. Kethiswaran , K. Brandes , et al. 2024. “Alteration of cGAS‐STING Signaling Pathway Components in the Mouse Cortex and Hippocampus During Healthy Brain Aging.” Frontiers in Aging Neuroscience 16: 1429005. 10.3389/fnagi.2024.1429005. 39149145 PMC11324507

[brb371130-bib-0085] Pietronigro, E. C. , V. Della Bianca , E. Zenaro , and G. Constantin . 2017. “NETosis in Alzheimer's Disease.” Frontiers in Immunology 8: 211. 10.3389/fimmu.2017.00211. 28303140 PMC5332471

[brb371130-bib-0086] Pluvinage, J. V. , M. S. Haney , B. A. H. Smith , et al. 2019. “CD22 Blockade Restores Homeostatic Microglial Phagocytosis in Ageing Brains.” Nature 568, no. 7751: 187–192. 10.1038/s41586-019-1088-4. 30944478 PMC6574119

[brb371130-bib-0087] Prabakaran, T. , C. Bodda , C. Krapp , et al. 2018. “Attenuation of cGAS‐STING Signaling Is Mediated by a p62/SQSTM1‐Dependent Autophagy Pathway Activated by TBK1.” The EMBO Journal 37, no. 8: e95402–e95402. 10.15252/embj.201797858. PMC5897779 29496741

[brb371130-bib-0088] Preeti, K. , A. Sood , V. Fernandes , I. Khan , D. K. Khatri , and S. B. Singh . 2024. “Experimental Type 2 Diabetes and Lipotoxicity‐Associated Neuroinflammation Involve Mitochondrial DNA‐mediated cGAS/STING Axis: Implication of Type‐1 Interferon Response in Cognitive Impairment.” Molecular Neurobiology 61, no. 9: 6217–6244. 10.1007/s12035-024-03933-y. 38285288

[brb371130-bib-0089] Rech, L. , and P. P. Rainer . 2021. “The Innate Immune cGAS‐STING‐Pathway in Cardiovascular Diseases—A Mini Review.” Frontiers in Cardiovascular Medicine 8: 715903. 10.3389/fcvm.2021.715903. 34381828 PMC8349977

[brb371130-bib-0090] Rocchi, A. , S. Yamamoto , T. Ting , et al. 2017. “A Becn1 Mutation Mediates Hyperactive Autophagic Sequestration of Amyloid Oligomers and Improved Cognition in Alzheimer's Disease.” PLos Genetics 13, no. 8: e1006962. 10.1371/journal.pgen.1006962. 28806762 PMC5570506

[brb371130-bib-0091] Rossi, B. , S. Angiari , E. Zenaro , S. L. Budui , and G. Constantin . 2011. “Vascular Inflammation in central Nervous System Diseases: Adhesion Receptors Controlling Leukocyte‐Endothelial Interactions.” Journal of Leukocyte Biology 89, no. 4: 539–556. 10.1189/jlb.0710432. 21169520

[brb371130-bib-0092] Saida, T. , M. Iima , R. Ito , et al. 2025. “Advances in Renal Cancer: Diagnosis, Treatment, and Emerging Technologies.” La Radiologia Medica 130, no. 10: 1540–1560. 10.1007/s11547-025-02066-z. 40751897

[brb371130-bib-0093] Scheltens, P. , B. De Strooper , M. Kivipelto , et al. 2021. “Alzheimer's Disease.” Lancet 397, no. 10284: 1577–1590. 10.1016/s0140-6736(20)32205-4. 33667416 PMC8354300

[brb371130-bib-0094] Shao, J. , Y. Meng , K. Yuan , et al. 2023. “RU.521 Mitigates Subarachnoid Hemorrhage‐Induced Brain Injury via Regulating Microglial Polarization and Neuroinflammation Mediated by the cGAS/STING/NF‐κB Pathway.” Cell Communication and Signaling 21, no. 1: 264. 10.1186/s12964-023-01274-2. 37770901 PMC10537158

[brb371130-bib-0095] Sharma, V. , R. Verma , P. Sharma , and T. G. Singh . 2025. “Therapeutic Modulation of cGAS‐STING Pathway in Neurodegeneration.” Brain Research 1864: 149784. 10.1016/j.brainres.2025.149784. 40517803

[brb371130-bib-0096] Shen, H. , C. Han , Y.i Yang , et al. 2021. “Pyroptosis Executive Protein GSDMD as a Biomarker for Diagnosis and Identification of Alzheimer's Disease.” Brain and Behavior 11, no. 4: e02063. 10.1002/brb3.2063. 33587329 PMC8035446

[brb371130-bib-0097] Shi, G. , L. Liu , Y. Cao , et al. 2023. “Inhibition of Neutrophil Extracellular Trap Formation Ameliorates Neuroinflammation and Neuronal Apoptosis via STING‐Dependent IRE1α/ASK1/JNK Signaling Pathway in Mice With Traumatic Brain Injury.” Journal of Neuroinflammation 20, no. 1: 222. 10.1186/s12974-023-02903-w. 37777772 PMC10543875

[brb371130-bib-0098] Song, Z. , K.‐W. Wang , H.‐T. C. Hagar , et al. 2024. “Hyperphosphorylated Tau Inflicts Intracellular Stress Responses That Are Mitigated by Apomorphine.” Molecular Neurobiology 61, no. 5: 2653–2671. 10.1007/s12035-023-03689-x. 37919601 PMC11043184

[brb371130-bib-0099] Srikanth, S. , J. S. Woo , B. Wu , et al. 2019. “The Ca(2+) Sensor STIM1 Regulates the Type I Interferon Response by Retaining the Signaling Adaptor STING at the Endoplasmic Reticulum.” Nature Immunology 20, no. 2: 152–162. 10.1038/s41590-018-0287-8. 30643259 PMC6340781

[brb371130-bib-0100] Standaert, D. G. , and G. M. Childers . 2022. “Alpha‐Synuclein‐Mediated DNA Damage, STING Activation, and Neuroinflammation in Parkinson's Disease.” Proceedings of the National Academy of Sciences 119, no. 17: e2204058119. 10.1073/pnas.2204058119. PMC9170025 35446614

[brb371130-bib-0101] Steinhagen, F. , T. Zillinger , K. Peukert , et al. 2018. “Suppressive Oligodeoxynucleotides Containing TTAGGG Motifs Inhibit cGAS Activation in human Monocytes.” European Journal of Immunology 48, no. 4: 605–611. 10.1002/eji.201747338. 29215161 PMC6386451

[brb371130-bib-0102] Takahashi, A. , T. M. Loo , R. Okada , et al. 2018. “Downregulation of Cytoplasmic DNases Is Implicated in Cytoplasmic DNA Accumulation and SASP in Senescent Cells.” Nature Communications 9, no. 1: 1249. 10.1038/s41467-018-03555-8. PMC5871854 29593264

[brb371130-bib-0103] Tan, H. W. S. , G. Lu , H. Dong , et al. 2022. “A Degradative to Secretory Autophagy Switch Mediates Mitochondria Clearance in the Absence of the mATG8‐Conjugation Machinery.” Nature Communications 13, no. 1: 3720. 10.1038/s41467-022-31213-7. PMC9240011 35764633

[brb371130-bib-0104] Tan, H. Y. , Y. K. Yong , Y. C. Xue , et al. 2022. “cGAS and DDX41‐STING Mediated Intrinsic Immunity Spreads Intercellularly to Promote Neuroinflammation in SOD1 ALS Model.” Iscience 25, no. 6: 104404. 10.1016/j.isci.2022.104404. 35712074 PMC9194172

[brb371130-bib-0105] Tesser, A. , G. M. Piperno , A. Pin , et al. 2021. “Priming of the cGAS‐STING‐TBK1 Pathway Enhances LPS‐Induced Release of Type I Interferons.” Cells 10, no. 4: 785. 10.3390/cells10040785. 33916318 PMC8067196

[brb371130-bib-0106] Thal, D. R. , and S. O. Tomé . 2022. “The central Role of Tau in Alzheimer's Disease: From Neurofibrillary Tangle Maturation to the Induction of Cell Death.” Brain Research Bulletin 190: 204–217. 10.1016/j.brainresbull.2022.10.006. 36244581

[brb371130-bib-0107] Tublin, J. M. , J. M. Adelstein , F. Del Monte , C. K. Combs , and L. E. Wold . 2019. “Getting to the Heart of Alzheimer Disease.” Circulation Research 124, no. 1: 142–149. 10.1161/circresaha.118.313563. 30605407 PMC6319653

[brb371130-bib-0108] Uddin, M. S. , M. M. Rahman , M. A. Sufian , et al. 2020. “Exploring the New Horizon of AdipoQ in Obesity‐Related Alzheimer's Dementia.” Frontiers in Physiology 11: 567678. 10.3389/fphys.2020.567678. 33584324 PMC7873563

[brb371130-bib-0109] Udeochu, J. C. , S. Amin , Y. Huang , et al. 2023. “Tau Activation of Microglial cGAS‐IFN Reduces MEF2C‐Mediated Cognitive Resilience.” Nature Neuroscience 26, no. 5: 737–750. 10.1038/s41593-023-01315-6. 37095396 PMC10166855

[brb371130-bib-0110] Vaillant‐Beuchot, L. , A. Mary , R. Pardossi‐Piquard , et al. 2021. “Accumulation of Amyloid Precursor Protein C‐Terminal Fragments Triggers Mitochondrial Structure, Function, and Mitophagy Defects in Alzheimer's Disease Models and Human Brains.” Acta Neuropathologica 141, no. 1: 39–65. 10.1007/s00401-020-02234-7. 33079262 PMC7785558

[brb371130-bib-0111] Van Acker, Z. P. , A. Perdok , R. Hellemans , et al. 2023. “Phospholipase D3 Degrades Mitochondrial DNA to Regulate Nucleotide Signaling and APP Metabolism.” Nature Communications 14, no. 1: 2847. 10.1038/s41467-023-38501-w. PMC10209153 37225734

[brb371130-bib-0112] Vincent, J. , C. Adura , P. Gao, et al. 2017. “Small Molecule Inhibition of cGAS Reduces Interferon Expression in Primary Macrophages From Autoimmune Mice.” Nature Communications 8, no. 1: 750. 10.1038/s41467-017-00833-9. PMC5622107 28963528

[brb371130-bib-0113] Wan, X. , H. Zhang , J. Tian , et al. 2024. “The cGAS‐STING/PERK‐eIF2α: Individual or Potentially Collaborative Signaling Transduction in Cardiovascular Diseases.” International Journal of Biological Sciences 20, no. 15: 5868–5887. 10.7150/ijbs.101247. 39664570 PMC11628330

[brb371130-bib-0114] Wang, B. , Y. Wang , J. Qiu , et al. 2023. “The STING Inhibitor C‐176 Attenuates MPTP‐Induced Neuroinflammation and Neurodegeneration in Mouse Parkinsonian Models.” International Immunopharmacology 124, no. Pt A: 110827. 10.1016/j.intimp.2023.110827. 37619411

[brb371130-bib-0115] Wang, Q. , S. Yuan , C. Wang , et al. 2024. “Brain Derived β‐Interferon Is a Potential Player in Alzheimer's Disease Pathogenesis and Cognitive Impairment.” Alzheimer's Research & Therapy 16, no. 1: 271. 10.1186/s13195-024-01644-z. PMC11662585 39709485

[brb371130-bib-0116] Wang, R. , and P. H. Reddy . 2017. “Role of Glutamate and NMDA Receptors in Alzheimer's Disease.” Journal of Alzheimer's Disease 57, no. 4: 1041–1048. 10.3233/jad-160763. PMC5791143 27662322

[brb371130-bib-0117] Wang, R. , Y. Zhu , Z. Liu , et al. 2021. “Neutrophil Extracellular Traps Promote tPA‐induced Brain Hemorrhage via cGAS in Mice With Stroke.” Blood 138, no. 1: 91–103. 10.1182/blood.2020008913. 33881503 PMC8288643

[brb371130-bib-0118] Wang, S. , L. Wang , X. Qin , et al. 2020. “ALDH2 Contributes to Melatonin‐Induced Protection Against APP/PS1 Mutation‐Prompted Cardiac Anomalies Through cGAS‐STING‐TBK1‐Mediated Regulation of Mitophagy.” Signal Transduction and Targeted Therapy 5, no. 1: 119. 10.1038/s41392-020-0171-5. 32703954 PMC7378833

[brb371130-bib-0119] Wang, W. , D. Hu , C. Wu , et al. 2020. “STING Promotes NLRP3 Localization in ER and Facilitates NLRP3 Deubiquitination to Activate the Inflammasome Upon HSV‐1 Infection.” PLoS Pathogens 16, no. 3: e1008335. 10.1371/journal.ppat.1008335. 32187211 PMC7080238

[brb371130-bib-0120] Wang, Y. , J. Luo , A. Alu , X. Han , Y. Wei , and X. Wei . 2020. “cGAS‐STING Pathway in Cancer Biotherapy.” Molecular Cancer 19, no. 1: 136. 10.1186/s12943-020-01247-w. 32887628 PMC7472700

[brb371130-bib-0121] Welch, G. M. , C. A. Boix , E. Schmauch , et al. 2022. “Neurons Burdened by DNA Double‐Strand Breaks Incite Microglia Activation Through Antiviral‐Like Signaling in Neurodegeneration.” Science Advances 8, no. 39: eabo4662. 10.1126/sciadv.abo4662. 36170369 PMC9519048

[brb371130-bib-0122] Wiser, C. , B. Kim , J. Vincent , and M. Ascano . 2020. “Small Molecule Inhibition of human cGAS Reduces Total cGAMP Output and Cytokine Expression in Cells.” Scientific Reports 10, no. 1: 7604. 10.1038/s41598-020-64348-y. 32371942 PMC7200739

[brb371130-bib-0123] Wu, H. , Y. Wang , W. Li , et al. 2019. “Deficiency of Mitophagy Receptor FUNDC1 Impairs Mitochondrial Quality and Aggravates Dietary‐induced Obesity and Metabolic Syndrome.” Autophagy 15, no. 11: 1882–1898. 10.1080/15548627.2019.1596482. 30898010 PMC6844496

[brb371130-bib-0124] Wu, L. , Y. He , B. Jiang , et al. 2016. “The Association Between the Prevalence, Treatment and Control of Hypertension and the Risk of Mild Cognitive Impairment in an Elderly Urban Population in China.” Hypertension Research 39, no. 5: 367–375. 10.1038/hr.2015.146. 26739869 PMC4865472

[brb371130-bib-0125] Wu, X. , N. Yu , Z. Ye , et al. 2023. “Inhibition of cGAS‐STING Pathway Alleviates Neuroinflammation‐Induced Retinal Ganglion Cell Death After Ischemia/Reperfusion Injury.” Cell Death & Disease 14, no. 9: 615. 10.1038/s41419-023-06140-0. 37726272 PMC10509212

[brb371130-bib-0126] Wu, Z. , W. Tang , F. E. E. M. Ibrahim , et al. 2023. “Aβ Induces Neuroinflammation and Microglial M1 Polarization via cGAS‐STING‐IFITM3 Signaling Pathway in BV‐2 Cells.” Neurochemical Research 48, no. 9: 2881–2894. 10.1007/s11064-023-03945-5. 37210413

[brb371130-bib-0127] Xia, M. , X. Li , S. Ye , et al. 2022. “FANCC Deficiency Mediates Microglial Pyroptosis and Secondary Neuronal Apoptosis in Spinal Cord Contusion.” Cell & Bioscience 12, no. 1: 82. 10.1186/s13578-022-00816-4. 35659106 PMC9164466

[brb371130-bib-0128] Xiao, Y. , C. Zhao , Y. Tai , et al. 2023. “STING Mediates Hepatocyte Pyroptosis in Liver Fibrosis by Epigenetically Activating the NLRP3 Inflammasome.” Redox Biology 62: 102691. 10.1016/j.redox.2023.102691. 37018971 PMC10106968

[brb371130-bib-0129] Xie, X. , G. Ma , X. Li , J. Zhao , Z. Zhao , and J. Zeng . 2023. “Activation of Innate Immune cGAS‐STING Pathway Contributes to Alzheimer's Pathogenesis in 5×FAD Mice.” Nat Aging 3, no. 2: 202–212. 10.1038/s43587-022-00337-2. 37118112

[brb371130-bib-0130] Xu, A. , Q. Zeng , Y. Tang , et al. 2020. “Electroacupuncture Protects Cognition by Regulating Tau Phosphorylation and Glucose Metabolism via the AKT/GSK3β Signaling Pathway in Alzheimer's Disease Model Mice.” Frontiers in Neuroscience 14: 585476. 10.3389/fnins.2020.585476. 33328854 PMC7714768

[brb371130-bib-0131] Xu, Q. , W. Xu , H. Cheng , H. Yuan , and X. Tan . 2019. “Efficacy and Mechanism of cGAMP to Suppress Alzheimer's Disease by Elevating TREM2.” Brain, Behavior, and Immunity 81: 495–508. 10.1016/j.bbi.2019.07.004. 31283973

[brb371130-bib-0132] Yan, M. , Y. Li , Q. Luo , et al. 2022. “Mitochondrial Damage and Activation of the Cytosolic DNA Sensor cGAS‐STING Pathway Lead to Cardiac Pyroptosis and Hypertrophy in Diabetic Cardiomyopathy Mice.” Cell Death Discovery 8, no. 1: 258. 10.1038/s41420-022-01046-w. 35538059 PMC9091247

[brb371130-bib-0133] Yang, K. , Z. Tang , C. Xing , and N. Yan . 2024. “STING Signaling in the Brain: Molecular Threats, Signaling Activities, and Therapeutic Challenges.” Neuron 112, no. 4: 539–557. 10.1016/j.neuron.2023.10.014. 37944521 PMC10922189

[brb371130-bib-0134] Yang, L. , C.‐C. Liu , H. Zheng , et al. 2016. “LRP1 Modulates the Microglial Immune Response via Regulation of JNK and NF‐κB Signaling Pathways.” Journal of Neuroinflammation 13, no. 1: 304. 10.1186/s12974-016-0772-7. 27931217 PMC5146875

[brb371130-bib-0135] Yang, N.‐S.‐Y. , W.‐J. Zhong , H.‐X. Sha, et al. 2024. “mtDNA‐cGAS‐STING Axis‐Dependent NLRP3 Inflammasome Activation Contributes to Postoperative Cognitive Dysfunction Induced by Sevoflurane in Mice.” International Journal of Biological Sciences 20, no. 5: 1927–1946. 10.7150/ijbs.91543. 38481801 PMC10929193

[brb371130-bib-0136] Yang, X. , L. Zhao , and Y. Pang . 2024. “cGAS‐STING Pathway in Pathogenesis and Treatment of Osteoarthritis and Rheumatoid Arthritis.” Frontiers in Immunology 15: 1384372. 10.3389/fimmu.2024.1384372. 38765007 PMC11099256

[brb371130-bib-0137] Yuan, H. , J. Yang , G. Qin , et al. 2025. “Regulation of STING G‐Quadruplex for Rescuing Cellular Senescence and Aβ Phagocytic Capacity of Microglia.” Chemical Science 16, no. 2: 693–699. 10.1039/d4sc04453c. 39634577 PMC11613991

[brb371130-bib-0138] Zenaro, E. , E. Pietronigro , V. D. Bianca , et al. 2015. “Neutrophils Promote Alzheimer's Disease‐Like Pathology and Cognitive Decline via LFA‐1 Integrin.” Nature Medicine 21, no. 8: 880–886. 10.1038/nm.3913. 26214837

[brb371130-bib-0139] Zeng, H. , Y. Gao , W. Yu , et al. 2022. “Pharmacological Inhibition of STING/TBK1 Signaling Attenuates Myeloid Fibroblast Activation and Macrophage to Myofibroblast Transition in Renal Fibrosis.” Frontiers in Pharmacology 13: 940716. 10.3389/fphar.2022.940716. 35924048 PMC9340478

[brb371130-bib-0140] Zhai, P. , Q. Chen , X. Wang , et al. 2024. “The Combination of Tanshinone IIA and Astragaloside IV Attenuates Myocardial Ischemia‐Reperfusion Injury by Inhibiting the STING Pathway.” Chinese Medicine 19, no. 1: 34. 10.1186/s13020-024-00908-y. 38419127 PMC10900662

[brb371130-bib-0141] Zhang, C. , G. Shang , X. Gui , X. Zhang , X. C. Bai , and Z. J. Chen . 2019. “Structural Basis of STING Binding With and Phosphorylation by TBK1.” Nature 567, no. 7748: 394–398. 10.1038/s41586-019-1000-2. 30842653 PMC6862768

[brb371130-bib-0142] Zhang, D. , Y. Liu , Y. Zhu , et al. 2022. “A Non‐Canonical cGAS‐STING‐PERK Pathway Facilitates the Translational Program Critical for Senescence and Organ Fibrosis.” Nature Cell Biology 24, no. 5: 766–782. 10.1038/s41556-022-00894-z. 35501370

[brb371130-bib-0143] Zhang, F. , D. Xian , J. Feng , et al. 2023. “Causal Relationship Between Alzheimer's Disease and Cardiovascular Disease: A Bidirectional Mendelian Randomization Analysis.” Aging 15, no. 17: 9022–9040. 10.18632/aging.205013. 37665672 PMC10522384

[brb371130-bib-0144] Zhang, H. , J. Ya , M. Sun , X. Du , J. Ren , and X. Qu . 2025. “Inhibition of the cGAS‐STING Pathway via an Endogenous Copper Ion‐Responsive Covalent Organic Framework Nanozyme for Alzheimer's Disease Treatment.” Chemical Science 16, no. 17: 7215–7226. 10.1039/d4sc07963a. 40144496 PMC11934151

[brb371130-bib-0145] Zhang, W. , G. Li , R. Luo , et al. 2022. “Cytosolic Escape of Mitochondrial DNA Triggers cGAS‐STING‐NLRP3 Axis‐Dependent Nucleus Pulposus Cell Pyroptosis.” Experimental & Molecular Medicine 54, no. 2: 129–142. 10.1038/s12276-022-00729-9. 35145201 PMC8894389

[brb371130-bib-0146] Zhang, X. W. , X. X. Zhu , D. S. Tang , and J. H. Lu . 2023. “Targeting Autophagy in Alzheimer's Disease: Animal Models and Mechanisms.” Zoological Research 44, no. 6: 1132–1145. 10.24272/j.issn.2095-8137.2023.294. 37963840 PMC10802106

[brb371130-bib-0147] Zhang, X. , J. Liu , S. Zhong , et al. 2025. “Exposure to Manganese Induces Autophagy‐Lysosomal Pathway Dysfunction‐Mediated Tauopathy by Activating the cGAS‐STING Pathway in the Brain.” Environmental Health 3, no. 2: 199–212. 10.1021/envhealth.4c00176. PMC11851216 40012869

[brb371130-bib-0148] Zhang, Z. , X. Yang , Y. Q. Song , and J. Tu . 2021. “Autophagy in Alzheimer's Disease Pathogenesis: Therapeutic Potential and Future Perspectives.” Ageing Research Reviews 72: 101464. 10.1016/j.arr.2021.101464. 34551326

[brb371130-bib-0149] Zhao, R 2024. “Exercise Mimetics: A Novel Strategy to Combat Neuroinflammation and Alzheimer's Disease.” Journal of Neuroinflammation 21, no. 1: 40. 10.1186/s12974-024-03031-9. 38308368 PMC10837901

[brb371130-bib-0150] Zhou, J. , Z. Zhuang , J. Li , and Z. Feng . 2023. “Significance of the cGAS‐STING Pathway in Health and Disease.” International Journal of Molecular Sciences 24, no. 17: 13316. 10.3390/ijms241713316. 37686127 PMC10487967

[brb371130-bib-0151] Zhu, L. , X.‐J. Hou , X.‐H. Che , et al. 2021. “Pseudoginsenoside‐F11 Attenuates Cognitive Dysfunction and Tau Phosphorylation in Sporadic Alzheimer's Disease Rat Model.” Acta Pharmacologica Sinica 42, no. 9: 1401–1408. 10.1038/s41401-020-00562-8. 33277592 PMC8379201

[brb371130-bib-0152] Zuo, Y. , S. Yalavarthi , K. Gockman , et al. 2020. “Anti‐Neutrophil Extracellular Trap Antibodies and Impaired Neutrophil Extracellular Trap Degradation in Antiphospholipid Syndrome.” Arthritis & Rheumatology 72, no. 12: 2130–2135. 10.1002/art.41460. 32729667 PMC7722115

